# The microRNA Let-7 and its exosomal form: Epigenetic regulators of gynecological cancers

**DOI:** 10.1007/s10565-024-09884-3

**Published:** 2024-06-05

**Authors:** Fei Wang, Chundi Zhou, Yanping Zhu, Maryam Keshavarzi

**Affiliations:** 1 Haiyan People’s Hospital, Zhejiang Province, Jiaxing, 314300 Zhejiang China; 2 School of Medicine, Tehran University of Medical Sciences, Tehran, Tehran Iran

**Keywords:** MicroRNA, Let-7, Gyncological cancer, Cervical cancer, Ovarian cancer, Endometrial cancer

## Abstract

Many types of gynecological cancer (GC) are often silent until they reach an advanced stage, and are therefore often diagnosed too late for effective treatment. Hence, there is a real need for more efficient diagnosis and treatment for patients with GC. During recent years, researchers have increasingly studied the impact of microRNAs cancer development, leading to a number of applications in detection and treatment. MicroRNAs are a particular group of tiny RNA molecules that regulate regular gene expression by affecting the translation process. The downregulation of numerous miRNAs has been observed in human malignancies. *Let-7* is an example of a miRNA that controls cellular processes as well as signaling cascades to affect post-transcriptional gene expression. Recent research supports the hypothesis that enhancing *let-7* expression in those cancers where it is downregulated may be a potential treatment option. Exosomes are tiny vesicles that move through body fluids and can include components like miRNAs (including let-7) that are important for communication between cells. Studies proved that exosomes are able to enhance tumor growth, angiogenesis, chemoresistance, metastasis, and immune evasion, thus suggesting their importance in GC management.

**Graphical Abstract:**

Impact of let-7 on female malignancies and diseases of the female reproductive tract. Let-7 expression is dysregulated in a variety of gynaecological and obstetric disorders.

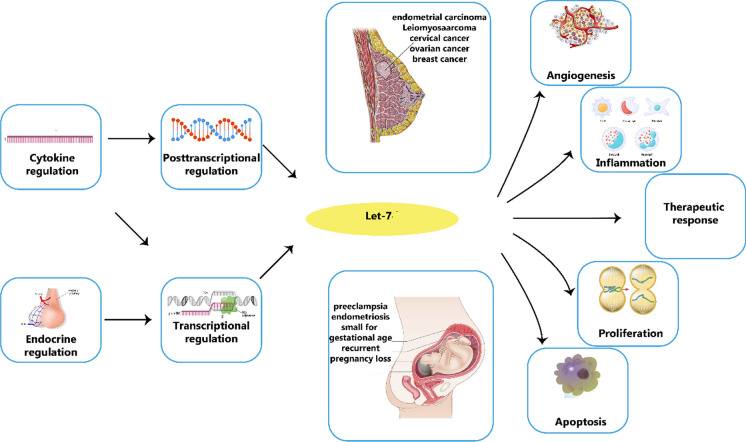

## Introduction

Gynecological cancers (GC) account for 12% of female cancers (Rema 2019). Gynecological cancers refer to cancers that originate in or spread to different organs related to reproduction, including the cervix, ovaries, uterus or endometrium, vagina, and vulva. GC generally develops in female reproductive organs situated within the pelvis. Each type of GC will have its own distinctive clinical presentation and susceptibility to certain genetic, environmental and lifestyle factors. GC becomes more common as women become older. In America, GCs affect over 90,000 women annually, and more than 28,000 of them die from the disease (Bourla and Zamarin 2016). The most common GCs, including ovarian, cervical, and endometrial malignancies are major concerns in women's health throughout the world (Zhang et al. 2019; Tian et al. 2021; Sun et al. 2021). Breast cancer is responsible for 32% of all cases among women. The predicted numbers of new cases and fatalities for breast cancer were determined to be 310,720 and 42,250, respectively. Despite being largely avoidable, cervical cancer is persistently responsible for the second highest number of women's deaths due to cancer, specifically in the age range of 20 to 39. This concerning trend has emerged in recent years and catapulted cervical cancer to the status of being the third most widespread cause of death in the young female population (Siegel et al. 2024). Although endometrial cancer (EC) is more common, the deadliest form of GC is ovarian cancer (OC) (Collins et al. 2014), and overall GC death rates are rising despite modern advances in diagnosis and treatment (Bourla and Zamarin 2016). The significant number of deaths related to GC emphasizes the urgency of conducting more studies and advancements in identifying it at an early stage and enhancing treatment options for late-stage GC.

MicroRNAs (miRNAs or miRs) have received much interest recently (Fattahi et al. 2023; Pordel et al. 2023; Rezaee et al. 2023) due to their capacity to control various biological functions. (Jonas and Izaurralde 2015; Safi et al. 2023). MicroRNAs are a group of tiny RNA molecules, approximately 22 nucleotides in length, that naturally occur within an organism and do not possess the ability to synthesize proteins (Mafi et al. 2022). Their main task is to control the degree of posttranscriptional gene expression (Bartel 2004; He and Hannon 2004; Ibrahim et al. 2014; Bartel 2018). The *let-7* (lethal-7) family, consisting of miRNAs, is highly prevalent and abundant in a wide range of animals, from worms to humans. (Bartel 2018; Letafati et al. 2022). The *let-7* family comprises numerous paralogous genes that are situated on distinct chromosomes (Büssing et al. 2008; Boyerinas et al. 2010; Roush and Slack 2008).

The *Let-7* miRNA family was initially identified as a crucial controller of differentiation in *C. elegans* worms (Copley et al. 2013; Reinhart et al. 2000). The mammalian Let-7 gene is often referred to as the "keeper of differentiation" since its aberrant expression and stimulation have been linked to cancer development (Büssing et al. 2008). Sequence similarity lends credence to the assumption that the roles of let-7 members are somewhat interchangeable (Brennecke et al. 2005). Repression of let-7 in cancer is often linked to a poor prognosis due to the fact that it targets many oncogenes (Balzeau et al. 2017). The let-7 family of miRNAs consists of thirteen members, each located on different loci spread across nine independent regions on seven distinct chromosomes (Wang et al. 2011). So far, scientists have identified 13 precursor genes that lead to the production of 10 distinct let-7 miRNAs in humans (miR-98, miR-202, let-7g, let-7f, let-7e, let-7d, let-7c, let-7b, and let-7a) (Sun et al. 2014). Due to their distinctive chromosomal locations, let-7 family members show varying degrees of transcriptional regulation (Chirshev et al. 2019). Research has demonstrated that the let-7 microRNA has the ability to control several crucial oncogenes, such as RAS, HMGA, c-Myc, and cyclin-D, resulting in the suppression of cancer growth, maturation, and advancement (Messina 2024).

## Biogenesis of Let-7

The maturation process of let-7 miRNAs involves stricter control in comparison to other miRNAs (Ali et al. 2020). In most cases, scientists have been able to identify a canonical mechanism for miRNA synthesis. The pathway can be divided into two clear stages. The initial step occurs in the nucleus and is carried out by two RNase III enzymes, Drosha and Dicer. The second step takes place in the cytoplasm, (Gregory et al. 2004; Lee et al. 2003; Hutvágner et al. 2001; Ketting et al. 2001). Nevertheless, deep sequencing has uncovered other subtypes of short RNA molecules that have a similar structure to conventional miRNAs but are generated by biogenesis pathways that omit one of the critical steps mentioned earlier. These recently identified miRNAs have been labeled as non-canonical (Berezikov et al. 2006; Cheloufi et al. 2010; Okamura et al. 2007; Ruby et al. 2007).

### Canonical miRNA biogenesis pathway

**Fig. 1:**
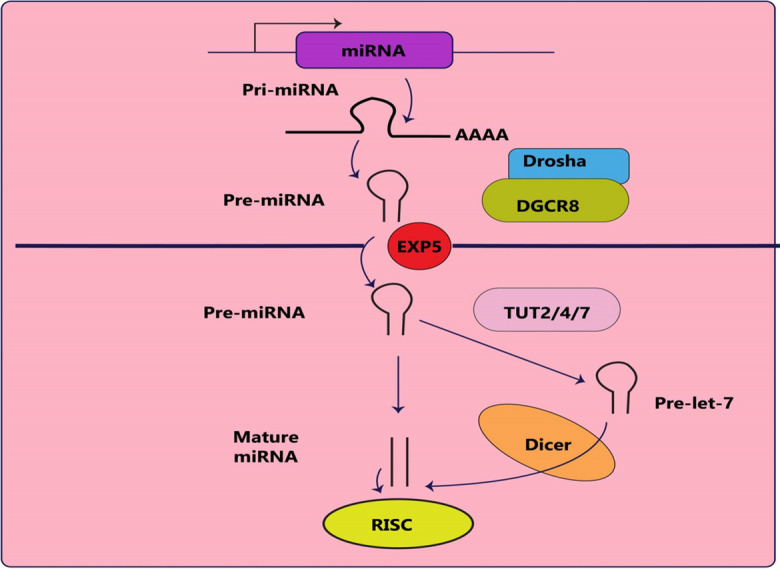
Canonical pathway of miRNA biogenesis. The standard miRNA biogenesis process is depicted in a schematic diagram. The Drosha microprocessor in the nucleus processes an RNA polymerase II-generated main miRNA transcript. An EXP5-Ran-GTP-dependent mechanism is utilized to transport the pre-miRNA to the cytoplasm for further processing by the Dicer microprocessor to produce a mature miRNA. Prior to Dicer-mediated processing, LIN28A and TUTases mono-uridylate pre-let-7 at its 3′ terminus. Translation of the target mRNA is prevented by loading the mature miRNA into the RISC

In the traditional process of miRNA synthesizes, Drosha and Dicer play crucial roles as microprocessors (Fig. 1). The appropriate genomic region is transcribed by RNA polymerase II to create a primary miRNA transcript, which has both a poly(A) tail at its 3′ end and a 5′ cap (Bracht et al. 2004). The primary microRNA consists of a stem-loop structure that contains approximately 33 base pairs (bp). Afterward, the pri-miRNA is cleaved by an enzyme called Drosha, which belongs to the RNase III family, and the protein DGCR8 (also known as Pasha), which has the ability to bind double-stranded RNA. (Gregory et al. 2004; Lee et al. 2003; Denli et al. 2004; Landthaler et al. 2004). The pri-miRNA holds an N6-methyladenylated GGAC sequence as well as other motifs. The distinctive hairpin shape of pri-miRNA is where Drosha cleaves the duplex at the base to form a pre-miRNA (Alarcón et al. 2015). As a result, the pre-miRNA will possess a 2 nucleotide 3' tail (Han et al. 2004). When the nuclear pore complex releases the combination of pre-miRNA, EXP5, and Ran-GTP, GTP is broken down, causing the pre-miRNA to detach from EXP5 and Ran-GTP and allowing it to separate into its individual pieces (Yi et al. 2003; Bohnsack et al. 2004; Lund et al. 2004).

The function of let-7 miRNA is regulated by a complex called miRNA-induced silencing complex (miRISC), which forms when one strand of the duplex guide is still attached to the RISC. The other passenger strand starts to degrade as a result of Argonaute RISC Catalytic Component 2 (Ago2) (Lee et al. 2016). The RISC employs the miRNA guide strand in order to bind to the 3'UTRs of mRNA through complementary base pairing, thereby controlling the post-transcriptional inhibition of the target genes of the miRNA. RISC causes the mRNA to degrade and undergo translational repression (Brennecke et al. 2005). Four times more mature miRNAs are stable when linked to AGO compared to mRNAs and can accumulate in cells up to as many as 500,000 copies (Reichholf et al. 2019).

Let-7-5p is a miRNA with 22 nucleotides produced by the enzyme Dicer (He and Hannon 2004). The fully developed let-7 miRNA possesses a double-helix shape in both primary and precursor forms. The mature let-7 miRNA (let-7-5p), also has a hairpin structure located in the stem, whereas let-7-3p miRNA, a partially complementary nucleotide strand, is found in the bud (Nam et al. 2011).

While the let-7 maturation process mostly follows the standard route for miRNA synthesis, for some let-7 family members, an additional step is required. Despite other members of the let-7 family all having a 3′ one-nucleotide overhang (pre-miRNAs in group II), only pre-let 7a-2, 7c, and 7e have the customary two-nucleotide 3′ overhang (pre-miRNAs in group I) (Heo et al. 2012). The group II precursors of pri-let-7, such as pri-let-7d, have an adenosine or uridine base in a bulge located close to the processing site (Heo et al. 2012). If the uridine/adenosine bulge is not detected by Drosha, a 3′ overhang composed of one nucleotide may be generated. The enzymes TUT2/PAPD4/GLD2, TUT4/ZCCHC11, and TUT7/ZCCHC6 are responsible for adding a single uridine to the 3′ end of group II pre-let-7s, resulting in a preferred two-nucleotide 3′ overhang recognized by Dicer (Heo et al. 2012).

### Non-canonical miRNA biogenesis pathway

**Fig. 2:**
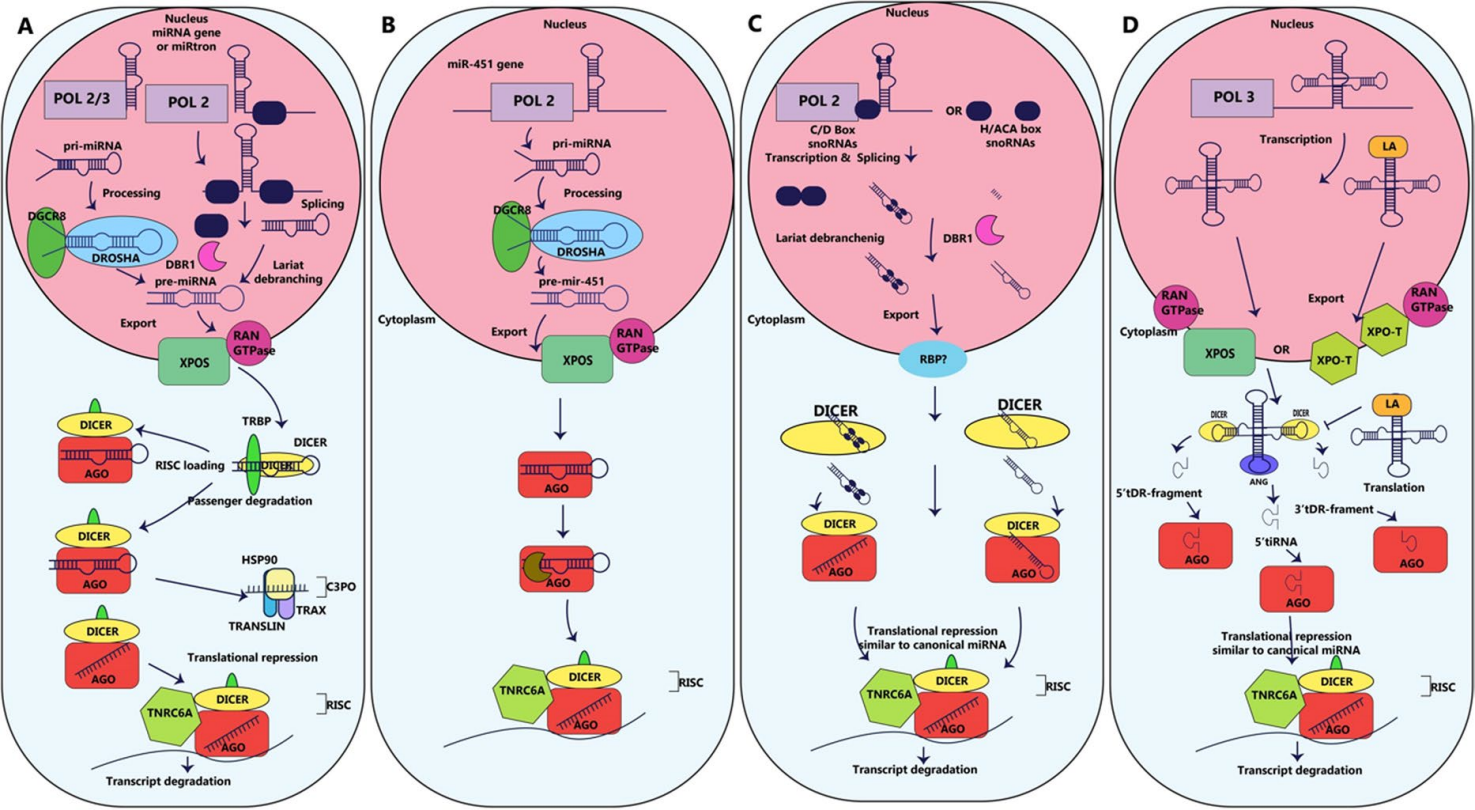
MicroRNA biogenesis (miRNAs). **A** POL II or POL III transcription of miRNA genes starts the canonical miRNA production. DROSHA/DiGeorge syndrome crucial region 8 processes pri-miRNAs (DGCR8). Exportin-5 (XPO-5) is a pre-miRNA transporter that moves them into the cytoplasm. Splicing out miRtrons and debranching the intron lariat by DBR1 creates pre-miRNAs. TRBP, a protein that cleaves exported pre-miRNAs, is part of the DICER/trans-activation-responsive RNA family. C3PO complex degrades the passenger strand. The RISC-loaded guide strand represses translation and degrades transcripts. **B** DICER-independent miR-451 processing. The passenger strand is cleaved and destroyed by AGO2-mediated cleavage following DROSHA/DGCR8 processing and export to the cytoplasm. **C** SnoRNAs are processed non-canonically to produce sdRNAs, which are RNAs derived from them. DBR1 debranches gene-spliced snoRNAs. Unknown mechanisms are involved in the export of SnoRNAs to the cytoplasm, where they are converted by DICER into sdRNAs and loaded into RISC. **D** MiRNAs are produced by non-canonical tRNA processing. XPO-5/XPO-T transfer tRNAs to the cytoplasm after transcription. The 5′ and 3′ loops are cut by DICER, releasing 5′ and 3′ tRNA-derived RNA (tDR) fragments. Angiogenin (ANG), which cleaves the anticodon loop (tiRNAs), is the source of 5′-tRNA stress-induced fragmentation. Every single tDR-fragment is loaded into RISC similarly to conventional miRNAs. (Bourla and Zamarin 2016) Lupus autoantigen (LA) stabilises after transcription, and XPO-T exports tRNAs to the cytoplasm. LA maintains tRNAs stablility for translation by inhibiting DICER processing. TNRC6A: trinucleotide repeat-containing gene 6A, HSP90: heat shock protein 90

Certain miRNAs, known as non-canonical miRNAs, have been discovered to originate by a distinct biogenesis process (Fig. 2). It has been suggested that non-canonical microRNAs play a role in several human health conditions, such as cancer (Patterson et al. 2017; Mo et al. 2019; Brameier et al. 2011). New insights into the mechanistic underpinnings of non-canonical miRNA biogenesis are discussed here.

For instance, MiRtrons are a specific group of non-traditional primary microRNAs that are found within the introns of coding genes (Berezikov et al. 2007). Mirtrons preferentially emerge from genes with larger numbers of introns. Firstly, unlike traditional pri-miRNAs, miRtrons are created using the nuclear splicing machinery as well as having a hairpin-like structure with a shorter stem. MiRtrons are also more stable than typical introns (Westholm and Lai 2011). Mirtrons are formed through a process that relies on splicing, without involving the enzyme Drosha. These unique types of microRNAs make up approximately 15% of all miRNAs found in the human body, which has captured the interest of scientists around the globe due to their atypical source, specific sequence features, evolutionary changes, and capability to regulate various cellular activities. They have also demonstrated great promise in the field of disease treatment (Salim et al. 2022). The debranching enzyme 1 (DBR1) performs lariat-debranching on these shorter hairpin configurations, because DROSHA/DGCR8 is unable to handle them (Okamura et al. 2007; Ruby et al. 2007). It has been found that cells with impaired DROSHA or DGCR8 activity retain intron-derived miRNAs (Ruby et al. 2007; Berezikov et al. 2007; Babiarz et al. 2008; Chong et al. 2010). Pre-miRNAs produced from miRtrons undergo the same processing steps as canonical miRNAs, including XPO-5 binding, transport into the cytoplasm, and DICER cleavage.

### Dicer-Independent miRNAs

Only one of the miRNAs found to exist has been suggested to be processed independently of DICER. To mature into a mature miRNA, as a result of its compact stem-loop structure, pre-miR-451 relies on the slicer activity of AGO2 in order to be cleaved, as DICER is unable to process it effectively (Cheloufi et al. 2010; Cifuentes et al. 2010). The GC concentration in the distal stem is low and the hairpin loop is long, along with the erroneous base pairing found in the stem are all required for pre-miR-451 processing, which is mediated by AGO2 (Yang et al. 2012a). Another RISC element is EIF1A, which initiates translation in eukaryotes, is necessary for the processing of miR-451 via AGO2 (Yi et al. 2015). Our understanding of pre-miR-451 processing has enabled the improvement of RNAi methodology. Improved RNAi can be facilitated because the AGO2 preferentially loads short interfering RNAs (siRNAs) thus enabling DICER-mutant tumour cells to target the expression of the specific genes (Herrera-Carrillo and Berkhout 2017).

### snoRNA-Derived miRNAs

The larger family of small nucleolar RNAs (snoRNAs) with a size range of 60–300 nt includes, Cajal-specific small nucleolar RNAs (sca-RNAs), snoRNAs with a H/ACA box (snoRAs, ACAs) and a snoRNAs with a C/D box (SNORDs) (Dupuis-Sandoval et al. 2015; Kufel and Grzechnik 2019). According to recent studies, snoRNAs target ribosomal RNA, which in turn affects gene expression in several ways (rRNA). Targeting rRNA sites by the 2′-O-methyltransferase Fibrillarin as well as the pseudouridylating enzyme Dyskerin are two snoRNA functions that are now well understood (Kufel and Grzechnik 2019; Meier 2017). There is also growing evidence that some snoRNAs can generate non-canonical miRNAs (Li et al. 2011; Ender et al. 2008; Scott et al. 2009). Interestingly, the stability and production of snoRNAs and snoRNA-derived miRNAs are influenced by the DICER and DGCR8 proteins, both crucial players in the traditional process for creating miRNAs. (Macias et al. 2012; Taft et al. 2009; Langenberger et al. 2013). The processing of miRNAs that originate from snoRNAs is affected by the ability of DGCR8 and other proteins to degrade these snoRNAs (Macias et al. 2015). SnoRNA-derived miRNAs, similar to canonical miRNAs, are around 21 nucleotides in length, bind to AGO1-AGO4, as well as suppress their target mRNAs (Brameier et al. 2011; Ender et al. 2008; Falaleeva et al. 2016).

### tRNA-Derived miRNAs

Transfer RNAs (tRNAs) are non-canonical miRNAs from another different source (Abdelfattah et al. 2014). tRNAs are made of a clover-leaf structure that DICER or Angiogenin (ANG) can cleave into pieces of tRNA-derived RNA (tDR) (Hasler et al. 2016; Li et al. 2018; Reinsborough et al. 2019). Additionally, recent information indicates that AGO proteins can carry specific tRNA fragments that control gene expression similarly to microRNAs (Hasler et al. 2016; Kumar et al. 2014).

Remarkably, RISC can be loaded with 3′ tDR-fragments caused by tRNA overexpression. This process is completely independent of DROSHA, XPO-5, and DICER (Kuscu et al. 2018). It has been demonstrated that these 3′ tDR-fragments can destabilize their target transcript stucture using RISC and the seed region (Kuscu et al. 2018). The factors that are involved in the creation of the 3′ tDR-fragments are still unknown. Because tRNAs are often overexpressed in human cancer, this process may be significant in these diseases (Zhang et al. 2018a).

### Nuclear biogenesis of miRNAs

**Fig. 3:**
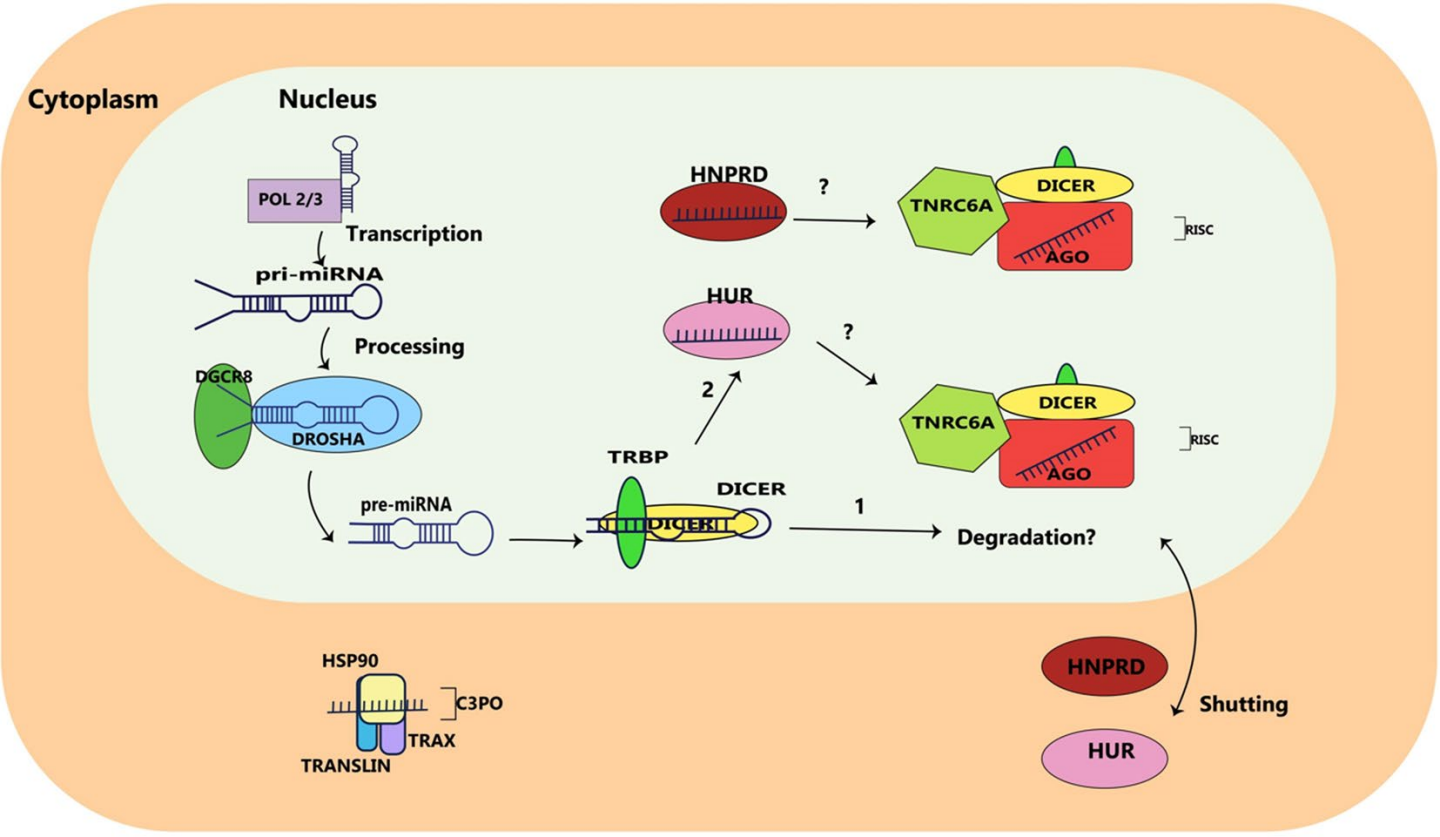
Mirna nuclear localisation. Pre-miRNAs undergo DICER/TRBP nuclear processing by DROSHA/DGCR8 for either (Rema 2019) destruction or (Bourla and Zamarin 2016) RISC loading by either human antigen R (HuR) or heterogeneous nuclear ribonucleoprotein D (HNRPD). This shuttles between the nucleus and the cytoplasm in an unclear manner

Human AGO1-4, TNRC6A and TRBP exist in the mammalian cell nucleus (Gagnon et al. 2014; Robb et al. 2005; Rüdel et al. 2008; Nishi et al. 2013). The AU-rich RNA-binding factor (AUF1), also known as HNRNPD or heterogeneous nuclear ribonucleoprotein D, is responsible for loading Let-7b onto AGO2, via one single RNA recognition motif (RRM) out of two (Yoon et al. 2015). HNRPD shuttles between the nucleus and cytoplasm (He and Schneider 2006) and might act as a potential RBP for RISC loading of certain miRNAs in the nucleus. Therefore, Let-7b and Let-7i can be loaded into AGO2 by the human antigen R (HuR) nucleus-and-cytoplasmic RBP (Fig. 3) (Yoon et al. 2013).

MiRNAs, a common class of small RNAs, have a substantial influence on regulating the expression of numerous messenger RNAs. They do so by either causing breakdown of the mRNAs or hindering their translation, or sometimes both. For this reason, they have a significant impact on the genetic expressions and protein compositions of eukaryotic organisms. In general, the creation of animal miRNAs involves two steps of processing, carried out by Drosha in the nucleus and Dicer in the cytoplasm, from lengthy primary transcripts containing at least one hairpin structure. Despite the established paradigm, there have been instances where deviations have been noticed: certain types of miRNAs do not entirely fit the traditional definition and are produced through alternative pathways, adding another layer of intricacy to the way miRNAs regulate gene expression.

## Let-7 and gynecological cancer

### Let-7 and ovarian cancer

#### Regulation of oncogenes

All AKT3, AKT2, and AKT1 are three protein kinase B isoforms that are controlled by the PI3K/AKT/mTOR pathway by tyrosine phosphorylation (Tian et al. 2021; Karege et al. 2010). Moreover, the significant development of epithelial ovarian cancer (EOC) is heavily influenced by the amplified activation and phosphorylation of AKT (Correa et al. 2012; Peart et al. 2012). In fact, AKT signaling is highly active in many types of human cancer. Hyper-activation of AKT is a characteristic of more than 50% of all human malignancies, and the upregulation of various AKT isoforms has been linked to numerous cancers in humans (Manning and Toker 2017). AKT1/2/3 are linked with each other in terms of their DNA sequence, but have been found to have distinct activities, particularly in cancer developement. On the other hand, ovarian cancer in mice with knockout of the AKT1 isoform results in smaller tumors and slower tumor growth (Linnerth-Petrik et al. 2016). The AKT1 kinase triggers a range of cellular signaling pathways that control cell survival and prevent apoptosis.

The protein AKT1 is an important objective for the treatment of cancer since its phosphorylation state is connected to decreased patient survival for malignancies other than EOC (Peart et al. 2012). Phosphorylation at T308 and S473 are critical regulatory sites for AKT1 activity. The kinase activity increased by 400-fold upon phosphorylation at T308 (Balasuriya et al. 2018). As well as regulating the substrate selectivity, phosphorylation at position S473 may additionally activate the kinase (Balasuriya et al. 2020). Insulin or growth factor-dependent phosphorylation increases AKT1 activity in cells. The phosphoinositide-dependent kinase (PDK1) phosphorylates T308 (Balasuriya et al. 2018; Alessi et al. 1997) and the mTORC2 complex (mammalian target of rapamycin) phosphorylates S473 (Manning and Toker 2017; Sarbassov et al. 2005). The inactivation of AKT1 occurs through the removal of phosphate groups from T308 by PP2A protein phosphatases and from S473 by PHLPP (PH domain leucine-rich repeat protein phosphatase) (Gao et al. 2005; Kuo et al. 2008). Several biochemical and cell-based studies have revealed how AKT1 activation works at a molecular level, however it is still unknown exactly which processes AKT1 controls or is controlled by.

Numerous miRNAs can target the AKT1 protein to regulate this protein in cancer cells. MiR-422a inhibits tumor development and cell proliferation in colorectal cancer via binding to the 3′-UTR of AKT1. In contrast, miR-422a down-regulation promotes tumor development by uncoupling the PI3K/AKT pathway from its normal regulatory mechanisms (Wei et al. 2017). In adult malignant glioma, according to research, when miRNA-637 is inhibited, it can no longer bind to the 3′-UTR of AKT1, resulting in an increased rate of cell invasion. Elevating the level of miR-637 had a contradictory impact by diminishing brain tumor cell proliferation, movement, and infiltration (Que et al. 2015). MiR-149 can suppress AKT1 expression in mesenchymal stem cells, and its loss promoted differentiation by upregulating AKT1 (Fan et al. 2022).

The ability of Let-7a to specifically target various oncogenes, including c-Myc, HMGA, Ras, STAT3, JAK, and NIRF which bear resemblance to ICBP90 and Np95, has been proven through research (Wang et al. 2012a). Let-7a homeostasis is tightly regulated, with abundant let-7a being necessary to avoid unchecked cell growth (Wang et al. 2012a). It has been found that let-7 miRNA inhibits temor development (Wang et al. 2012a).

**Fig. 4:**
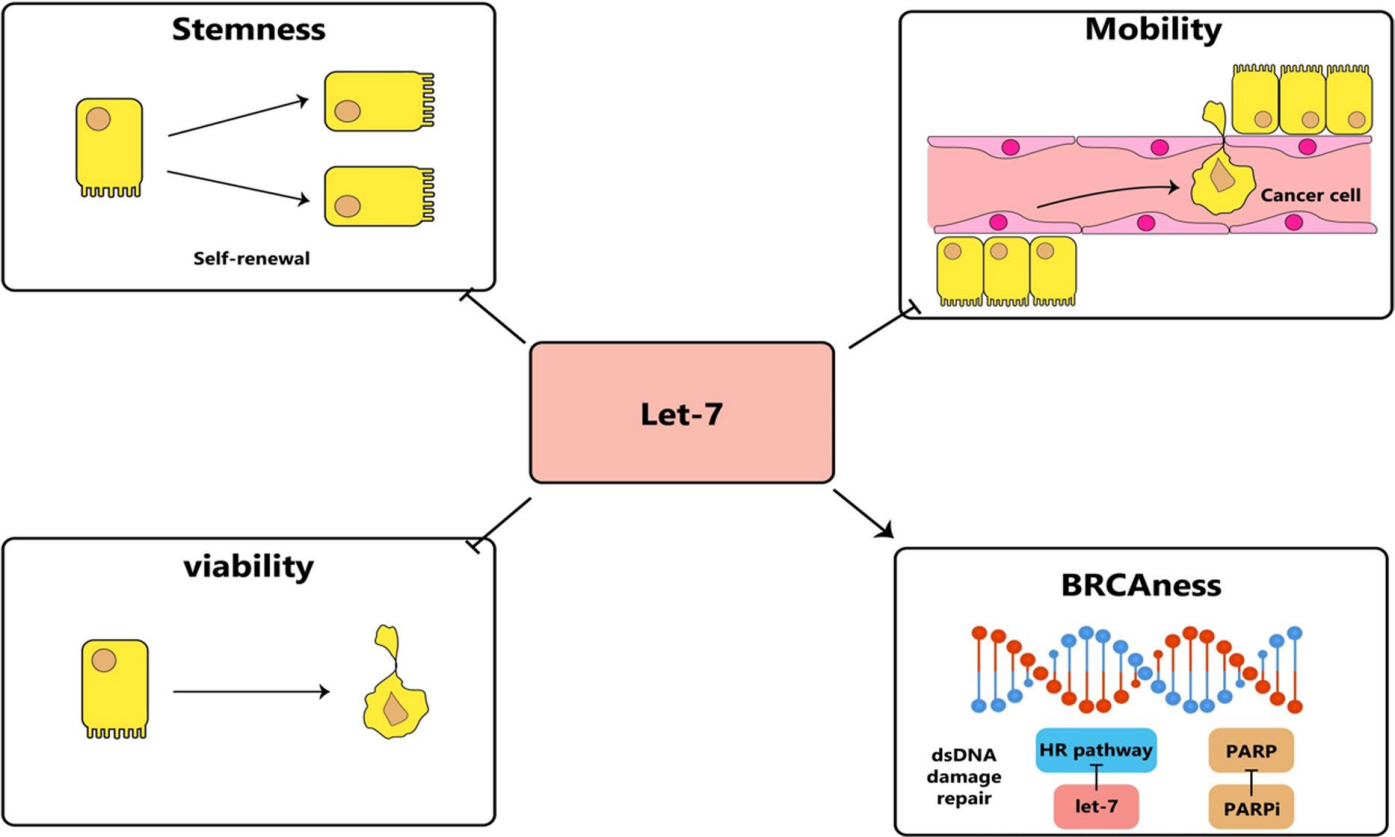
Role of let-7 in ovarian cancer

T308 phosphorylation was shown to be much lower in EOC spheroids, as reported by Frederick et al., while let-7 miRNA levels were found to be elevated with the switch from adherent cell growth to spheroids (Frederick et al. 2022). They looked at how let-7 miRNA can alter the phosphorylation state and cellular activity of AKT1 using molecular investigations. In HEK 293T cells activated by growth factors and treated with let-7a, AKT1 phosphorylation was upregulated at threonine 308 and downregulated at serine 473, while the phosphorylation of the downstream AKT1 substrate GSK-3 was increased. Deregulation of AKT signaling was produced by both let-7b and let-7g because they may render AKT1 insensitive to growth factor stimulation. The deregulation of PI3K pathway components that regulate AKT1 phosphorylation and activity, including PI3KC2A, PDK1, and RICTOR, was shown to be let-7a dependent. Together, their results revealed a novel function for miRNAs in controlling AKT signaling (Frederick et al. 2022). Figure 4 shows the function of let-7 in ovarian cancer.

#### Regulation of pathways

Homologous recombination DNA repair (HRR) is a process in which the healthy gene copy serves as a pattern for repairing the damaged DNA as a treatment for cancer patients who have a homologous recombination DNA-repair deficit (HRD), either through somatic mutations in the tumor itself. or through germline mutation carrier status. BRCA1/2 genes are responsible for repairing damaged DNA, and relative somatic or germline mutations may leak to HRD. PARPi inhibitors interfere with nucleus-based DNA-repair mechanisms. Human cells with HRD are unable to repair DNA utilizing HR, thus leading to cell death (Gupta et al. 2019). In addition to BRCA1/2 mutations, susceptibility to PARPi may be caused by abnormalities in HRR. The name for this characteristic is "BRCAness" (Byrum et al. 2019). Under identical clinical conditions with the same PARPis treatment, response rate and survival of HRD patients were higher than those without HRD (Coleman et al. 2017; Moore et al. 2019; González-Martín et al. 2019; Mirza et al. 2016). In the SOLO2 study, BRCA mutation carriers who had ovarian cancer that responded to platinum-based treatment were given the PARP inhibitor olaparib as a form of maintenance therapy, resulting in a 13-month improvement in overall survival after receiving two or more cycles of platinum-based treatment (Poveda et al. 2021). In individuals without BRCA1/2 mutations, inducing the BRCAness phenotype could enhance progression-free survival and sensitivity to targeted drug therapy, possibly resulting in a longer overall survival. When discussing the value of germline and somatic tumor diagnostics, PARPi has become the classical drug type that could benefit from this information. The combination of bevacizumab and maintenance therapy, as well as monotherapy for patients with heavily pre-treated recurrent disease, result in advantageous outcomes (Falzone et al. 2021; Jiang et al. 2019). Many experiments have been performed to test the effectiveness of PARPi in conjunction with immune therapy, VEGF inhibitors, and other targeted therapies. One particular example is the attention that cediranib (a tyrosine kinase inhibitor for PDGF and VEGF) has received (An et al. 2021). Increased levels of pluripotency, and self-renewal at a cellular level, and greater tumor burden were seen in patients with low let-7 levels (Chirshev et al. 2020). Hence, therapeutic let-7 replacement may be an approach for inhibiting cancer cells. The Overexpression of let-7, a microRNA known for its tumor-fighting abilities, effectively represses the expression of a multitude of cancer-causing genes. Some of these oncogenes, LIN28A and HMGA2, are markers of CSCs and have been correlated with cancer development, progression, and chemotherapy resistance (Büssing et al. 2008; Zhou et al. 2013). Embryonic stem cells (ESCs) produce the pluripotency factor LIN28A, and LIN28A levels decrease when ESCs differentiate into somatic cells (Parisi et al. 2017). Cancers with high LIN28A expression generally show a poor prognosis, and LIN28A suppression by exogenous let-7 slowed tumor growth in mice (He et al. 2018; Albino et al. 2016).

#### Interactions with other regulatory factors

HMGA2 is a protein that is connected to chromatin and alters its structure by binding to areas containing a high amount of A/T base pairs, ultimately aiding in the activation of gene expression. During embryonic development, HMGA2 is present in large amounts; however, it is not found in mature cells. Its role is crucial in converting dormant embryonic stem cells into precursor cells (Parisi et al. 2017; Navarra et al. 2016). Early differentiation is dependent on HMGA2 and the escape of ESCs from pluripotency (Navarra et al. 2016). The observed effects of variations in let-7 sequences could be attributed to changes in target specificity among the 13 members of the let-7 family (Chirshev et al. 2019). The various members of the let-7 family could potentially have conflicting impacts on tumor development, as certain members may act to suppress tumors while others may act as promoters or agents that catalyze the growth of cancerous cells (Wang et al. 2018; Brueckner et al. 2007; Shi et al. 2017). Many factors, including BRCA1, PARP, RAD51, E2F1, and IGF1, have been linked to the suppression effect of let-7 on DNA repair (Chirshev et al. 2019; Wang et al. 2018; Wielgos et al. 2017; Huang et al. 2017; Shen et al. 2014). Tumor suppressor effects of let-7i were shown by Chirshev et al., including increased apoptosis, inhibition of invasion and migration, and decreased cell stemness, all of which processes are critical for cancer development, recurrence, and metastasis (Chirshev et al. 2021). The quality of exhibiting BRCAness was elevated due to the overexpression of let-7i, as evidenced by the fact that tumors without BRCA mutations displayed heightened sensitivity to the PARP inhibitor olaparib. Additionally, the HRR pathway is inhibited by let-7i, pointing to a possible method of inducing the BRCAness phenotype. For these reasons, let-7i has been evaluated as a potential therapeutic miRNA for EOC, especially in patients whose cancers lack HRR pathway mutations (Chirshev et al. 2021). Surgery and chemotherapy are effective first-line treatments for most patients with HGSOC (high-grade serous ovarian cancer) (70–80%). Unfortunately, 70% to 90% of patients suffer a recurrence, leading to a poor prognosis and disappointing survival rate (Hoppenot et al. 2018). Based on different morphologies and molecular markers, several tumor subtypes have recently been grouped under the HGSOC umbrella (Konecny et al. 2014; Coleman et al. 2013; Tothill et al. 2008; Tan et al. 2013). It is expected that improved treatment response rates and survival outcomes could result from a more nuanced categorization of tumor heterogeneity, which would allow for the use of precision medicine approaches (Bedard et al. 2013). To prevent the spread of chemo-resistant tumor clones at the metastatic stage, some targeted treatments are being investigated and developed (Vaughan et al. 2011). After successful primary treatment, it is believed that tumour recurrence is triggered, at least in part, by the persistence of just few cancer stem cells (CSC). This explains the recurrence and metastasis of tumors after potentially curative primary treatment (surgery and chemotherapy) (Beck and Blanpain 2013). Improved prognostic indicators are essential for the develoipment of more effective treatment methods (Agarwal and Kaye 2005). The HGSOC molecular subtypes called C1 (mesenchymal) and C5 (proliferative or StemA) have been linked to increased platinum resistance and poor prognosis, respectively (Tothill et al. 2008; Tan et al. 2013; Verhaak et al. 2013). A certain subpopulation of cancer cells exhibits properties of both epithelial and mesenchymal cell types. CSC features and a poor prognosis is linked to this 'hybrid' phenotype (Grosse-Wilde et al. 2015; Strauss et al. 2011). In part, there is a connection between SNAI1, which is similar to the Drosophila Snail protein, and various aspects such as the induction of epithelial mesenchymal transition (EMT), the maintenance of stem cell properties, resistance to chemotherapy, and the invasive nature of high-grade serous ovarian cancer (HGSOC) (Hojo et al. 2018; Lu et al. 2012a; Kurrey et al. 2009). Tumors may be classified based on whether or not they have an epithelial or mesenchymal cell type. It has been established that miRNAs are abnormally produced in HGSOC, and have an effect on cisplatin-induced apoptosis (Yang et al. 2008a; Yang et al. 2012b; Wang et al. 2013; Sorrentino et al. 2008). Let7 is essential for controlling stem cell development both in worms and in humans, and let-7 improves their capacity for self-renewal (Copley et al. 2013; Hayes and Ruvkun 2006). Let7 targets several proteins that regulate cell cycle progression, control pluripotency, and repair DNA damage (Boyerinas et al. 2010). The variety of target genes that are expressed in any particular cell determines how let7 dysregulation manifests itself. Due to the continued activity of let-7 in somatic cell differentiation, repression of Let7 is required for achieving pluripotency in somatic cells (Unternaehrer et al. 2014). The potential of let-7 as a biomarker is highlighted in its role in predicting the prognosis of ovarian cancer (Yang et al. 2008b). According to Chirshev et al., expression of let-7 was inversely linked with stemness, suggesting that loss of let-7 is a characteristic of the cancer stem cell phenotype (Chirshev et al. 2020). Lower levels of let-7 were discovered to be linked to a lower EMT score, increased spheroid growth and cancer development, and higher susceptibility to platinum-containing anti-cancer drugs. Surprisingly, The concept of stemness was not linked to invasiveness, as evidenced by the fact that cells with low levels of let-7 demonstrated greater migratory abilities. The researchers reached the conclusion that let-7 expression levels were closely connected to in vitro self-renewal, the epithelial-mesenchymal transition (EMT) state, and the amount of tumor present in vivo (Chirshev et al. 2020).

On the other hand, a decrease in the level of expression of let-7a, let-7e, and let-7f, in addition to let-7, was associated with the potential for invasiveness in EOC tumors. One of the most important miRNAs is miR-34a which regulates p53, and inhibits tumor development by suppressing proliferation and survival (Iorio and Croce 2013; Zhang et al. 2015). The miR-200 family members play a crucial role in regulating the EMT and their down-regulation significantly impacts this function (Zhang et al. 2008a). The Let-7 molecule has been shown to have a suppressive effect on tumors, as evidenced by the fact that reduced levels of this molecule have been associated with a negative outlook in almost all types of human cancers (Wang et al. 2012a). Cancer development, differentiation, and progression are all suppressed by let-7's ability to inhibit oncogenic proteins such as RAS (Wang et al. 2012a; Shell et al. 2007; Johnson et al. 2007a), HMGA2 (Johnson et al. 2007a; Lee and Dutta 2007; Peng et al. 2008; Yu et al. 2007; Mayr et al. 2007), c-Myc (Wang et al. 2012a; Shell et al. 2007; Johnson et al. 2007a), and cyclin-D2 (Wang et al. 2012a; Shell et al. 2007). It has been proposed that the let-7 family contains numerous potential oncogenes. Brueckner et al. and others have demonstrated that let-7a overexpression is linked to a more aggressive cancer phenotype in humans (Brueckner et al. 2007). A connection has been demonstrated between the abnormal expression of let-7 members and the development of chemotherapy drug resistance (Yang et al. 2008b; Boyerinas et al. 2012; Xiao et al. 2017; Cai et al. 2013). In 2008, it was proposed that the let-7i expression profile could be used to categorize EOC patients into those who would respond favorably to cisplatinum-based chemotherapy and those who would not. Even though this phenomenon is clearly influenced by the let-7i targets, the exact targets responsible for this remain unknown (Yang et al. 2008b). Further studies by Xiao et al. showed that let-7e affected the DNA double-strand break repair pathway to enhance the response of EOC cells to cisplatin (Xiao et al. 2017).

The different behavior of different members of the extensive let-7 family in cancers arising in different bodily organs necessitates a deeper comprehension of the connection between distinct types of cancer and let-7 microRNAs. Biamonte et al. investigated the function of let-7 g in EOC. They studied the impact of artificially increasing let-7 g levels in OVCAR3 and HEY-A8 E cells, which resulted in the following outcomes: (i) decrease in c-Myc and cyclin-D2 levels, leading to cell cycle arrest; (ii) reduced expression of Slug, snail, and inhibition of EMT; and (iii) heightened responsiveness to cis-platinum (chemosensitization) (Biamonte et al. 2019). The expression of let-7 g was then compared in human EOC tumor and non-tumor tissues, and it was discovered that let-7 g was substantially lower in EOC tissue samples (*p* = 0.0002). The minimal tissue levels of let-7 g in patients with advanced EOC significantly increased the risk of developing chemoresistance (*n* = 17, *p* = 0.03194). This conclusion was supported by an analysis of serum samples taken from the same patients (*n* = 17, *p* = 0.003). After thorough investigation, researchers determined that let-7 g plays a crucial role in inhibiting the development of EOC tumors, making it a potential treatment option for those who do not respond to cisplatin-based therapy. The researchers proposed that decreased levels of let-7 g in both tissues and serum could serve as an indicator for the development of chemoresistance among EOC patients. (Biamonte et al. 2019).

The pluripotency-associated RNA-binding protein LIN28 inhibits let-7 biogenesis by preventing it from being processed to its mature form. Nevertheless, lower LIN28 expression levels were seen in differentiated cells (Viswanathan et al. 2008). It should be noted that let7 post-transcriptional regulation by LIN28 works in conjunction with transcriptional control of this miRNA family to determine overall levels (Lee et al. 2016). The transcription of let-7 is also thought to be controlled by TWIST1, a transcription factor implicated in the EMT, as well as TP53, NFKB1, MYC, BMI1, and CEBPA (Chirshev et al. 2019). By examining how LIN28, let-7, and the miRNA-200 family are cross-regulated, a theory was developed linking EMT to stemness (Jolly et al. 2014). There have been a few studies showing links between loss of differentiation and EMT transcription factors. After it was found that let-7i was directly inhibited by TWIST1 (Yang et al. 2012c), Yang et al. asked whether let-7i was similarly potentially suppressed by SNAI1, another EMT transcription factor. Inhibiting the EMT in cancer stem cells may be a key tactic for enhancing patient prognosis. There is evidence from numerous studies that connect the EMT to the emergence of stem cell traits (Battula et al. 2010). EMT transcription factors, specifically SNAI1, TWIST1, SNAI2, and ZEB1, have a profound impact on the increase in cell population possessing stem cell properties (Mani et al. 2008; Wellner et al. 2009; Morel et al. 2008; Bhat-Nakshatri et al. 2010). Recent studies showed that a hybrid state is maintained by SNAI1 expression in cells (Kröger et al. 2019). A computational biology approach showed that SNAI2 could perform the same activity (Subbalakshmi et al. 2021). Therefore hybrid EMT cells are more likely to become stem cells (Jolly et al. 2014; Kröger et al. 2019). Repressing CDH1 and other epithelial factors, stimulating mesenchymal factors, and repressing microRNAs like miR-34 are all examples of the SNAI1 transcription factor activity (Siemens et al. 2011). Additionally, let-7 miRNA potentially interacts with SNAI1. Loss of let-7, which may bind to SNAI1 promoters, and its early overexpression during reprogramming are connected (Unternaehrer et al. 2014). One possible method involves using antisense oligonucleotides to target genes such as those involved in the EMT. Some technological problems faced by such methods include allowing extravasation and cellular absorption by specific cells without activating the immune system or being degraded by ubiquitous nucleases (Wittrup and Lieberman 2015). Mesoporous silica nanoparticles (MSN) with a size of 50–200 nm, have a high surface area because of their porous nature (Lu et al. 2007a). They can be loaded with siRNA cargo by being coated with cationic polyethyleneimine (PEI), and The absorption of substances by particular cells can be improved by conjugation with hyaluronic acid (HA) (Shahin et al. 2018; Hom et al. 2010). Ovarian cancer stem cells have CD44 overexpressed on their surface, thus binding the ligand HA on their surface and increasing the cell uptake (Zhang et al. 2008b). One study by Wang et al. investigated the molecular basis for EMT-induced cancer stemness (Wang et al. 2021). The EMT factor SNAI1 has been linked to let-7 suppression and acquisition of stemness in stem cell reprogramming. It is not fully known how let-7 is lost in cancer cells. Using spheroid formation, patient-derived xenografts, miRNA, mRNA, and protein expression, they investigated stem cell phenotype and tumor progression in 4 cancer cell lines from pancreatic, ovarian and breast tumors. On the other hand, studies have proven that when EMT-inducing growth factors are introduced, or if there is an overexpression of SNAI1, the depletion of SNAI1 results in a decrease in stemness and a reversal of let-7 expression. Let-7 is a mediator of the pro-stemness effects of SNAI1, according to in vitro rescue studies. SNAI1 knockdown using siRNA delivered by nanoparticles in orthotopic patient-derived xenografts, resulted in decreased stemness, higher let-7 expression, and lower tumor burden in vivo. Both luciferase and chromatin immunoprecipitation assays revealed that let-7 promoters were bound by SNAI1, thus inhibiting let-7 transcription. The stemness pathways for cancer cells involved the SNAI1/let-7 axis according to their findings, and this could be further investigated as a potential treatment target directed at CSCs (Wang et al. 2021). Here is a list of published research articles that discuss the significance of let-7 in relation to ovarian cancer as shown in Table 1.

**Table 1:** Research articles that discuss the significance of let-7 in relation to ovarian cancer

Expression status	Target	Model	Type of cell line	Ref
Upregulated	GSK-3β	In vitro	HEK 293T and COS-7 cells	Frederick et al. 2022)
Upregulated	LIN28A and HGMA2	In vitro	OVCAR8 and NCCIT	Chirshev et al. 2021)
Upregulated	OCT4, NANOG, LIN28A, and HMGA2	In vivo and In vitro	OVCAR8, FTSEC, and NCCIT	Chirshev et al. 2020)
Upregulated	ABC transporters, ErbB, RAS and HIF-1 pathways	In vitro	sKOV-3	García-Vázquez et al. 2018)
Downregulated	HMGA2 and P53	In vitro	SKOV3	Yang et al. 2012d)
Downregulated	IMP-1 MDR1 HMGA2Actin	In vitro	NCI/ADR-RES, OVCAR8, T47D, IGROV1,,LOX-IMVI, HEK-293, and SKOV3ip	Boyerinas et al. 2012)
Downregulated	Cyclin-D2 and c-Myc	In vitro	HEY-A8 and OVCAR3	Biamonte et al. 2019)
Downregulated	*CCND1*, *CDC25A*, *HMGA2*, *IL6* and *LIN28B*	In vivo and In vitro	A2780, 2008, and HOSE	Wang et al. 2012b)
Downregulated	HMGA2, LIN28B and IGF2BP1	In vitro	45 OC cell lines, ES-2, HEK293A, and MCF-7	Busch et al. 2016)
Downregulated	(*Hmga2*, *c-Myc* and *Igf2bp3)*	In vivo (tissue) and In vitro	A2780, Tara R127, ARK2, and HEK293	Yan et al. 2015)
Downregulated	Lin-28B and Lin-28	In vivo	-	Lu et al. 2009)
Downregulated	PGRMC1	In vitro	SKOV-3	Wendler et al. 2011)
Downregulated	*HMGA2*, *LIN28*, *LIN28B*, *MYC*, *MYCN*, *DICER1*, and *RNASEN*	In vivo	-	Helland et al. 2011)
Downregulated	HMGA2, SNAI1, α/β-TUBULIN, LIN28A	In vivo and In vitro	OVSAHO, OVCAR8, HEK293T, PANC-1, MCF-7	Wang et al. 2021)
Upregulated	IGF-II or IGFBP-3	In vivo	-	Lu et al. 2007b)
Upregulated	GSK-3β, AKT1, PI3KC2A, PDK1, RICTO, and PI3K	In vitro	HEK 293T, COS-7, and TOV-21G	Frederick et al. 2022)
Upregulated	Snail, E-cadherin, α/β-tubulinand, and GAPDH	In vivo and In vitro	OVCAR8 and COV318, OVSAHO, Kuramochi, D2F, and NCCIT	Hojo et al. 2018)
Downregulated	BRCA1 and Rad51	In vitro	A2780, HO8910, ES2, CAOV3, SKOV3, and OV2008	Xiao et al. 2017)
Downregulated	GSK-3β and p53	In vitro	BG-1, UCI-101, and HCT116	Guo et al. 2013)
Downregulated	MPO, WT1, Pax-8, PDPN	In vivo	-	Johnson et al. 2020)
Downregulated	HMGA2E, cadherin, vimentin, Snail, and Actin	In vitro	SC1 and SC2	Shell et al. 2007)
Downregulated	*HMGA2, C14orf28, HMGA2, LIN28B, ARID3B, HIC2, KIAA1196*	In vivo (tissue) and In vitro	BG-1, UCI-101, HEY, OVCA420, OVCAR2, OVCA432, OVCA433, OVCAR3, OVCAR5, and HOSE-B	Dahiya et al. 2008)
Downregulated	HMGA2	In vitro	SC1 and SC2	Park et al. 2007)
Downregulated		In vivo		Lu et al. 2011a)
Downregulated	KLK6 and KLK10	In vitro	OVCAR-3	Bayani et al. 2013)
downregulation	Lin28b, PSP94, and prostasin	In vivo and In vitro	HIO-80, Ovca432, Ovca433, Ovcar3, and Caov3	Ma et al. 2014)
Downregulated	CDC34	In vitro	AGS, HCT116, Colo320, MCF7, HepG2, K562, NT2, and PA1	Kim et al. 2012)
Downregulated	KRAS	In vivo	-	Ratner et al. 2010)
Downregulated	HMGA2	In vitro	T29H, SKOV3, HEY, OVCAR-3, and Caov-3	Xi et al. 2014)
Upregulated	Dicer1, miR-152, and RAD51	In vivo (tissue) and In vitro	C13, OV2008, A2780, A2780/DDP, HO8910, SKOV3, CaOV3, Hey, and COV362	Wang et al. 2018)
Downregulated	LIN28	In vitro	A2780, T47D, MCF7, and HeLa	Zhong et al. 2010)
Downregulated	KRAS	In vivo (tissue) and In vitro	Caov3	Kim et al. 2014)
Downregulated	FST,CCNF, CDC25A, PLAGL1, and ARID3A	In vitro	SVOG3e	Wang et al. 2015a)
Upregulated	FOXL2	In vitro	KGN and COV434	Rosario et al. 2013)
Downregulated	Hmga2, c-Myc, and Igf2bp3	In vivo (tissue) and In vitro	A2780, Tara R127, ARK2 and HEK29314	Yan et al. 2015)

### Let-7 and cervical cancer

**Fig. 5:**
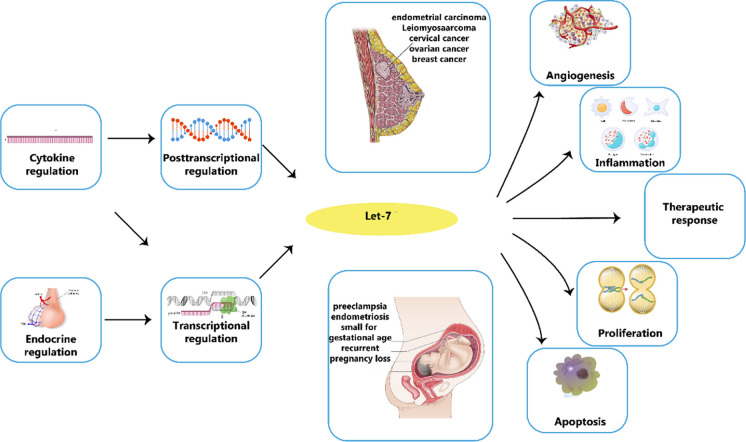
The influence of let-7 on cancers and illnesses affecting the female reproductive system is significant. Let-7 expression is disrupted in a range of obstetric and gynecological disorders. This disruption is caused by hormonal and cytokine factors and is controlled both during transcription and after. The importance of let-7 in the progress and management of various medical conditions lies in its ability to target key mRNA molecules involved in regulating cellular growth, apoptosis, angiogenesis, and immune cell function

The let-7 family of miRNAs is present in numerous species and serves as a direct counterpart to the let-60/RAS gene (Reinhart et al. 2000; Johnson et al. 2005). The identical seed sequence (5'-GAGGUAG-3') of Let-7 miRNAs has led to the belief that they target the same transcripts, a notion that has been held for a significant period of time (Johnson et al. 2005). It has been discovered that let-7 miRNAs target many oncogenes and regulatory genes, such as RAS (Johnson et al. 2005), HMGA2 (Yu et al. 2007), Cyclin D1/2/3 and Cyclin A (Schultz et al. 2008), IL6 (Iliopoulos et al. 2009), c-MYC (Sampson et al. 2007), DICER1 (Tokumaru et al. 2008), LIN28B and LIN28A (Guo et al. 2006). A few of the crucial biological processes that the Let-7 family targets include, self-renewal, proliferation, cell cycle regulation, pluripotency, inflammation and tumor suppression. The stem of the let-7 miRNAs contains the 5' sequence (5p), which has a large number of matches with the partially complementary 3' sequence (3p), and there is a terminal loop region with a variable length and structure that connects them. Both the pri-let-7 and the pre-let-7 miRNAs also can bind to this structure. RNA-binding proteins dock at the pre-element (preE) of pre-let-7 miRNAs. Examples of these proteins are LIN28A/LIN28B, TRIM25, hnRNPA, and KHSRP (also referred to as KSRP) (Nam et al. 2011; Viswanathan et al. 2008; Heo et al. 2008; Michlewski and Cáceres 2010; Newman et al. 2008; Rybak et al. 2008; Trabucchi et al. 2009). The predetermined process of creating miRNAs (also known as Group I) produces three specific let-7 miRNAs (let-7a-2, let-7e, and let-7c), whereas other let-7 miRNAs (classified as Group II) need extra steps for their formation due to a one-nucleotide extension at the 3' end. This is related to the fact that some pri-let-7 sequences contain an additional bulge near the processing site, which hinders the microprocessor complex from properly recognising and cleaving the pri-miRNA (Heo et al. 2012). The diagram in Fig. 5 showcases the significant impact of let-7 on a range of female diseases and conditions related to the reproductive system, encompassing cervical cancer, ovarian cancer, and endometrial cancer.

#### Interaction with LIN28 proteins

Proteins called LIN28A and LIN28B that bind to RNA are structurally and functionally related members of the LIN28 family, which is substantially conserved across species. Let-7 and LIN28A were both first identified in *C. elegans* (Horvitz and Sulston 1980). Numerous cancer types, most notably cervical cancer, have been documented to overexpress LIN28A, however, undifferentiated stem cells are where overexpressed LIN28A is most frequently seen (Viswanathan et al. 2009). Let-7, LIN28A/LIN28B, and target mRNAs have shown an opposite conection in expression between normal and malignant tissues, suggesting that they might have opposite effects on how cancer develops and spreads (Viswanathan et al. 2009; Shyh-Chang and Daley 2013). The functional correlation between LIN28B/LIN28A and let-7 has been widely studied (Wang et al. 2015b). The CCHC-type zinc finger (ZnF12) domains and the N-terminal cold-shock domain (CSD) within LIN28A and LIN28B function to inhibit the Let-7 miRNAs. Both the LIN28B and LIN28A proteins have the ability to attach to the let-7 pre-E domain, thus impeding the progress of Drosha in the nucleus and Dicer in the cytoplasm in their processing tasks (Thornton et al. 2012). The pre-let-7 3' overhang is monouridylated by the uridyl-transferases TUT2, TUT4, and TUT7, allowing Dicer to recognize it and synthesize a mature let-7 when LIN28A expression is absent (Heo et al. 2012). Pre-let-7, on the other hand, is degraded by the 3'-5' exonuclease Dis3L2 following an interaction with LIN28A that triggers the recruitment of TUT7 or TUT4 to lengthen the 3' terminus with approximately 14 uracyl residues (Hagan et al. 2009; Heo et al. 2009a; Chang et al. 2013). LIN28B may regulate let-7 in the nucleus since it contains nuclear and nucleolar localization signals (Boyerinas et al. 2010). Although only sharing 70% similarity with LIN28A, LIN28B contains the same CSD and ZnF12 domain sequences, which might facilitate interaction with let-7 in the same manner as LIN28A does (Nam et al. 2011; Loughlin et al. 2011; Heo et al. 2009b). While the translocation mechanism for LIN28A has been identified, it has been hypothesized that LIN28B may also inhibit cytoplasmic let-7 synthesis through the TUT4 and Dis3L2 pathway (Lui et al. 2007). LIN28A interacts with many let-7 miRNAs, and their molecular basis has been extensively studied.

Certain sequences of LIN28A, including the CSD and ZnF12 domains, have been shown to interact with preE. The 5'-NGNGAY-3' motif in a loop (preE-bulge) and the ideal preE are produced by the 5'-NGNNG-3' sequence (preE-stem), which are separated by a spacer of varying length, and dock with the CSD and interact with ZnF12 (Nam et al. 2011; Heo et al. 2009a; Loughlin et al. 2011). According to this hypothesis, the preE-loop wraps around the CSD extension, making several interactions with the RNA loop (Nam et al. 2011).

Several types of cancer seem to have different regulatory patterns for various let-7 miRNAs. When it comes to miRNAs, cervical cancer shows a general downregulation but the upregulation of certain specific mature let-7s (Lui et al. 2007; Lee et al. 2008). Both LIN28B and LIN28A may inhibit let-7 miRNAs to a comparable extent (Nam et al. 2011; Loughlin et al. 2011), and there is evidence that they accomplish this via interacting with let-7 precursors and binding to the preE structure (Loughlin et al. 2011; Sharma and Mohanty 2017). Whether or not these variations are indicative of cellular regulatory alterations that cause cancer remains unknown. To control the production of let-7, LIN28 proteins bind to certain sites in the pre-miRNA structure (Zamora-Contreras and Alvarez-Salas 2018). Nevertheless, new evidence suggests that some let-7 miRNAs can avoid being regulated by LIN28. One study examined The correlation between the levels of LIN28B expression and the presence of pre-let-7 miRNAs in different cervical cell lines with varying levels of malignancy and human papillomavirus (HPV) loads. RTqPCR was used to quantify levels of let-7a in the cells. Immunoblotting was used to examine let-7 effects on LIN28B and other targets. Computational techniques were used to compare let-7 and LIN28B expression levels as well as the sequences and structures of let-7 precursors (prelet-7).

After studying multiple cell lines, it was determined that all of them contained noticeable amounts of Lin28B protein. However, among these lines, the highest levels were found in the ones derived from tumors. Nearly every cell line displayed elevated pre-miR-98 levels and pre-let-7c/f-1, regardless of their malignancy or expression of LIN28B. None of the cell lines had pre-let-7e and pre-let-7-a3, but pre-let-7a-2 had extensive presence, while pre-let-7g/i showed mainly in expression in tumor cell lines. Pre-miR-98 and pre-let-7i/g/f-1 were found to have a positive association with LIN28B in tumor cell lines. Analysis and sequence alignment of pre-let-7 miRNAs suggested that structural characteristics inside the preE area might affect the optimal pre-let-7 structure for interacting with LIN28B. The pre-let-7 sequence included short preE-stems that may bypass LIN28B regulation, but lengthy preE-stems were often linked to abundant miRNAs in the gene (Zamora-Contreras and Alvarez-Salas 2018). PreEs in various cell lines have different architectures, which in turn affects their ability to interact with LIN28B and regulate let-7 in a manner that is not shared by all lines. The variability of pre-let-7 levels among various cervical cell lines can be attributed to this factor. Previous studies have revealed that certain single nucleotide polymorphisms (SNPs) in the miRNA promoter region have been associated with the development and/or outcome of various cancers, including cervical cancer. (Xie et al. 2013; Shen et al. 2015; Sui et al. 2016; Liang et al. 2015; Wang et al. 2015; Chen et al. 2015; Li et al. 2013; Pan et al. 2015; Yuan et al. 2016). According to Liang et al., a positive correlation was found between the rs4705343 C > T mutation in the promoter of miR-143/145 as well as the likelihood of developing cervical squamous cell carcinoma (CSCC). Reduced transcriptional activity has been seen for the rs4705343C allele (Sun et al. 2017). For instance, Yuan et al. demonstrated a connection between an increased likelihood of developing cervical cancer and rs4938723 C > T in the pri-miR-34b/c promoter (Yuan et al. 2016).

#### Polymorphisms in the let-7 promoter

Recently, Xie et al. studied the correlation between two SNPs and the onset of hepatocellular carcinoma (Xie et al. 2013). The rs10877887C variation was shown to significantly increase the chance of death in both patients with stage B liver cancer at the Barcelona clinic, as well as those who had received chemotherapy (Xie et al. 2013). More recent research has examined these two SNPs in various human disorders, including cancers of the lung (Shen et al. 2015), thyroid (Wang et al. 2015), liver (Sui et al. 2016), and brain (Sima et al. 2015), along with major depressive disorder (Liang et al. 2015). Liu et al.'s research looked at whether there was a correlation between the likelihood of developing CSCC and polymorphisms in the let-7 promoter, at the rs10877887 and rs13293512 loci (Liu and Ni 2018). 358 healthy individuals acted as controls, whereas 331 CSCC patients had been diagnosed. Additionally, rs10877887 genotyping was done using RFLP PCR. For the rs13293512 genotyping, Taqman allelic discrimination was employed. The let-7 family expression was examined via qPCR. Those with the rs10877887CC genotype had a greater likelihood of developing CSCC in comparison to those with the rs10877887TT or rs10877887 TT/CT genotypes (adjusted OR = 2.11, 95% CI, 1.34–3.31, p0.002). When comparing the risk of CSCC for the rs10877887T and rs10877887C alleles, the adjusted OR was 1.35 with a 95% confidence interval of 1.08 to 1.69 and a p-value of 0.008. When compared to people with the rs10877887CT/TT + rs13293512CT/TT genotype, individuals with the rs10877887CC + rs13293512CC genotype had a 4.78-fold higher chance of having CSCC (OR = 4.78, 95% CI, 1.78–12.84, p0.001). There was also a significant decrease in let-7i in CSCC tissues from patients with the rs10877887CC genotype (*p* = 0.02). From these results, it seems that rs10877887 could serve as a biomarker for detecting CSCC development (Liu and Ni 2018). Prior research has combined genomic and molecular data with the HPV status (Integrated genomic and molecular characterization of cervical cancer 2017), but stage-specific transcriptomic analysis profiling has not yet been conducted. Patients diagnosed with CC have a 65% chance of surviving for five years if it is caught early, but this number can potentially be improved by identifying early warning signs and prognostic markers in addition to diagnostic biomarkers. Based on the latest research, cervical cancer is believed to be caused by being infected with high-risk viruses such as HPV types 16 and 18. These viruses contain cancer-causing genes at both the initial and later stages, including E1, E2, E6, E7 and E6. E6 is responsible for regulating the breakdown of p53, a protein that protects against tumors, and E7 triggers the release of E2F by activating pRb, leading to increased cell growth. This collaboration between the two kinases is often deemed to be a significant factor in the development of cervical cancer (Tomaić 2016). The expression of the human papillomavirus and host genes may be affected by epigenetic alterations, which have been found in several genes such as MGMT, FHIT, RASSFA1, CDK, E-cadherin, APC, ER1, etc., at different stages (Lu et al. 2012b) of CC (Sen et al. 2018).

#### LncRNAsas a sponge for miRNAs

MALAT1, EBIC, HOTAIR, and GAS5 are only a few of the lncRNAs that affect CC carcinogenesis, while many more are dysregulated in gynecological malignancies (Hosseini et al. 2017; Peng et al. 2016). In several cases, researchers discovered that specific long non-coding RNAs (lncRNAs) were found to play a significant role in altering the metastatic potential of cells by functioning as a highly effective absorbent for microRNAs, consequently diminishing their capacity to bind to specific messenger RNAs (mRNAs). (Olgun et al. 2018). ANRIL has been shown to interact with miR-381 (Zhang et al. 2018b), while GAS5 has been shown to inhibit miR-196A and miR-205 (Yang et al. 2017), and DLEU1 has been shown to interact with miR-381 (Liu et al. 2018). Because of their ability to regulate the production of mRNA targets, the potential of several miRNAs is being investigated in the prognosis, therapy, and diagnosis of CC (Sadri Nahand et al. 2019; Banno et al. 2014). There have been reports that several miRNAs that have the potentiel for diagnosis of CC, including miR-29a, miR-10a, miR-21, miR-20b, miR-16, miR-9, miR-106, miR-125, miR-34a, and miR-375 (Pardini et al. 2018). Other miRNAs could be prognostic markers for CC including miR-188, miR-99a, miR-223, and miR-125b (Gao et al. 2018), miR-145, and miR-196 (Chen et al. 2017). By utilizing current databases and datasets, cutting-edge biomarker selection techniques, and modern bioinformatic tools to investigate CC samples at different stages, it is possible to create a comprehensive collection of non-coding RNA panels (Bolha et al. 2017), of which miRNAs and lncRNAs could be very useful cancer biomarkers (Fardi et al. 2023; Huang et al. 2023; Aghaei-Zarch 2024; Huang and Aghaei-Zarch 2024; Shirvani et al. 2022). This study suggested an approach that aims to thoroughly extract and analyze RNA-based biomarkers as a means of gaining deeper insight into the disrupted pathways and RNA molecules associated with cervical cancer. This was linked with the clinical evaluation as the cornerstone of the International Federation of Gynaecology and Obstetrics (FIGO) staging (Banerjee and Karunagaran 2019). After the patient staging process, data from the public domain were mined to find relevant RNAs. After determining which miRNA families were particularly important by analysis of interaction networks between mRNAs and miRNAs and lncRNAs, they generated an association network between mRNA, miRNA, and lncRNA that was tumor stage-specific. The selected lncRNAs and mRNAs underwent integrated bioinformatic analysis. There was a correlation between the expression of Stage I markers: SLC2A1, HBA1, HBA2, HBA2, HBB, CXCL10, stage III marker: PKIA, and stage IV marker: S100A7. Among the selected miRNA partners, the let-7 family miRNAs were overrepresented. According to DSigDB research, a combination of collagen fibrils and other multimeric structures, progesterone CTD 00006624, were the most crucial markers in the R-HSA-2022090 pathway for *Homo sapiens* (Banerjee and Karunagaran 2019). The best performing survival markers were shown to be hsa-miR-188-3p, SLC2A1, hsa-miR-150-5p, and hsa-miR-378a-3p (Banerjee and Karunagaran 2019). The frequency of lncRNA dysregulation in many different cancer types shows that the development of cancer may be significantly affected by abnormal lncRNA expression (Gibb et al. 2011). Various forms of human cancers including breast, hepatocellular, and bladder cancers show upregulation of the 2.3-kb lncRNA H19. This result raises the possibility that H19 plays an oncogenic role (Matouk et al. 2007). Epigenetic activation of the miR-200 family in hepatocellular carcinoma has been shown to prevent H19-mediated metastasis, indicating that H19 may have tumor-suppressive activity (Lee et al. 2015; Yoshimizu et al. 2008; Zhang et al. 2013). Extensive amounts of the long non-coding RNA HOTAIR (Antisense RNA for the HOX gene) have been associated with a heightened likelihood of developing breast cancer. Research indicates that HOTAIR plays a significant role in promoting the spread and invasion of cancer by disrupting the structure and organization of chromatin (Gupta et al. 2010; Wu et al. 2015a). In addition, it has been discovered that the carcinogenic lncRNA MALAT1 (Transcript 1 for metastasis-associated lung adenocarcinoma) acts as a decoy for splicing factors, thus causing splicing failure (Tripathi et al. 2010).The competitive endogenous RNA (ceRNA) theory, put forth by Leonardo et al., proposes that RNA transcripts that contain MREs may distinguish miRNAs from other targets that also have MREs, hence regulating the production of those miRNAs (Ebert et al. 2007; Salmena et al. 2011; Tay et al. 2014). It follows that mRNAs, transcribed pseudogenes, circRNAs, and lncRNAs, all of which include MREs, may regulate each other in this manner (Salmena et al. 2011). There is evidence that any transcript containing one or more MREs could function as a ceRNA (Yang et al. 2016). The mRNAs encoded by CeRNAs could potentially play a part in numerous biological pathways (Karreth et al. 2011). These ceRNAs control where the miRNAs can bind to their targets, adding another layer of post-transcriptional control. There is strong evidence that lncRNAs and miRNAs compete with each other for binding, affecting the degree to which their targets are de-repressed. In hepatocellular cancer, miR-9 is sequestered by the long non-coding RNA HULC, which de-represses PPARA and initiates the ACSL1-related positive feedback loop. As a result, the ceRNA activity of HULC generates a complicated auto-regulatory loop (Cui et al. 2015). It was discovered by Liu et al. (Guo et al. 2017), that in cervical cancer tissues, Ras suppressor protein 1 pseudogene 2 (RSU1P2) acts as a lncRNA which is abundantly expressed and promotes tumor progression. Competition for let-7a is one mechanism for this regulation. This competition fuels the aggressiveness of cervical cancer cells. The transcription factor N-myc increases the oncogenic potential of RSU1P2 by promoting its own expression through a self-reinforcing loop. Cancer treatment approaches that specifically target RSU1P2 may have significant benefits (Guo et al. 2017). Cervical cancer is still a worldwide health concern, although Pap smears and HPV DNA tests used in screening programs have been effective in lowering the number of new cases and deaths caused by certain diseases. (Yim and Park 2007). Therefore, numerous efforts have been undertaken to identify dependable prognostic biomarkers for the emergence of cervical intraepithelial lesions, but as yet to no avail. Since many human cancers affect miRNA expression, miRNA expression profiles are likely to be effective markers for cancer detection (Pereira et al. 2010).

#### Dysregulation in cervical cancer

Specific types of small RNA molecules called microRNAs are produced within abnormal areas of the cervix known as cervical intraepithelial lesions and in cases of cervical cancer (Pereira et al. 2010; Lv et al. 2019). Let-7c is one of 13 closely related miRNAs that have a conserved genomic location and are commonly lower in human malignancies (Calin et al. 2004). Let-7c is downregulated in a variety of malignancies, including cervical carcinoma (Lv et al. 2019; Lui et al. 2007; Ma et al. 2012). Furthermore, let-7c expression is known to be dysregulated after HPV infection, according to previous research (Nuovo et al. 2010). A single research analyzed the manifestation of let-7c in exfoliated cells from individuals with cervical intraepithelial lesions as a potential indicator for HPV-induced cancer (Malta et al. 2015). To analyze the levels of let-7c expression, a qPCR was employed on samples taken from 73 female individuals, including both those with healthy tissue and those with cervical intraepithelial lesions. Their study included 38 individuals with normal epithelium, of which 17 individuals had HPV infection and 21 individuals did not, 14 individuals with low grade squamous intraepithelial lesions, as well as 21 individuals with high grade squamous intraepithelial lesions. Low-grade squamous intraepithelial lesions in patients were associated with down-regulation of let-7c (2(-Ct) = 0.38, *p* = 0.06) and high grade lesions showed reduced let-7c expression (2(-Ct) = 0.21, *p* = 0.004). Let-7c expression was shown to be downregulated in all cervical intraepithelial lesions (2(-Ct) = 0.27; *p* = 0.011). Let-7c expression was shown to be considerably altered across a spectrum of cervical intraepithelial lesions, suggesting future study as a potential biomarker for CIL utilizing exfoliated cervical cells as a sample source (Malta et al. 2015). Table 2 lists some published research articles about the function of let-7 in cervical cancer.

**Table 2:** Some published research articles about the function of let-7 in cervical cancer

Expression status	Target	Model	Type of cell line	Ref
Downregulated	LIN28B, c-MYC and RAS	In vitro	SiHa, QGH,CaSki, QGU, HeLa, C-33A, CxT1, Cx16.2, HEPG2, and HKc16E6/E7II	Zamora-Contreras and Alvarez-Salas 2018)
Downregulated	-	In vivo	-	Liu and Ni 2018)
Downregulated	miR-let-7c-5p/IGF-1R/PI3K/AKT and -catenin/SLUG		HeLa	Shao et al. 2020)
Downregulated	RSU1P2, IGF1R, N-myc, and EphA4	In vivo and In vitro	HeLa and C33A	Guo et al. 2017)
Downregulated	-	In vivo	-	Malta et al. 2015)

### Let-7 and endometrial *cancer*

#### DICER1 mutations

DICER1 is an endoribonuclease in charge of miRNA synthesis, and a crucial gene acting as a cancer-driving factor in endometrial cancer (Bailey et al. 2018). Endometrial adenocarcinoma samples exhibited an increased number of hotspot biallelic DICER1 mutations, based on the TCGA PanCancer and MSK-IMPACT (Memorial Sloan Kettering Integrated Mutation Profiling and Actionable Cancer Targets) databases (Vedanayagam et al. 2019). The pre-miRNA stem loop is split into two mature miRNAs by DICER1 (Bartel 2004; Wang et al. 2017a). Modifications in the processing of miRNA have been linked to the development of endometrial cancer, while ovarian cancers are often accompanied by hotspot mutations in DICER1 (Wang et al. 2015a; Vedanayagam et al. 2019; Chen et al. 2015; Heravi-Moussavi et al. 2012; Anglesio et al. 2013; Gurtan et al. 2012). The myometrial invasion was similarly linked to lower DICER1 expression, higher FIGO-grade tumours, and endometrial cancer recurrence (Wang et al. 2017b; Torres et al. 2011; Zighelboim et al. 2011).

Using the PgrCre/ + ; Dicer1flox/flox mouse model, Dicer1 deletion in the uterus led to a uterine phenotype that was comparable to the atrophic endometrium in postmenopausal women, showing no glandular epithelium, a single layer of luminal epithelium, and a minimal to nonexistent endometrial stroma (Hawkins et al. 2012). At least 80% of endometrial adenocarcinomas experience the loss or mutation of the tumor suppressor phosphatase and tensin homolog (PTEN) (Tashiro et al. 1997). In the mice with PTEN-deleted uterus (PgrCre/ + ; Ptenflox/flox) 88.9% showed well-differentiated endometrial cancers developed by day 90 (Daikoku et al. 2008). DICER1 is thought to have a substantial role in endometrial adenocarcinoma even though loss of PTEN is a feature of this cancer (Wang et al. 2020). Dicer1 and PTEN-deficient mice developed endometrial adenocarcinomas that were poorly differentiated and overexpressed the clear-cell adenocarcinoma markers, HNF1B (hepatocyte nuclear factor 1 homeobox B), and napsin A. These adenocarcinomas did not respond to hormones. In humans, treatment with progesterone had no impact on the growth of poorly-differentiated adenocarcinoma or adnexal metastases. When DICER1 was knocked out, different transcriptome patterns and broad miRNA downregulation were observed in mouse uterine samples or Ishikawa cells. This was the first weakly differentiated endometrial cancer model in genetically engineered mice (Wang et al. 2020). Computational integration with mRNA targets was used to demonstrate the dysregulation of genes (Let-7 and miR-16 target). This was in line with previously reported endometrial cancer samples showing the human DICER1 mutation from the TCGA (The Cancer Genome Atlas). Several distinct pathways responsible for signaling, such as the transforming growth factor-beta and ephrin-receptor pathways, are not functioning properly in tumors, specifically those in human endometrial cancers. LIM kinase 2 (LIMK2), is an essential part of p21 cell signaling and raises the possibility of a hormone-independent etiology for endometrial cancer. Endoribonuclease Dicer1 is necessary for the synthesis of most miRNAs. The pairing of the two RNase III domains of Dicer1 is capable of creating an internal dimer structure, which in turn facilitates the precise cutting of the pre-miRNA hairpin and the generation of fully developed miRNAs. These miRNAs may interact with their respective target mRNAs in a miRNA-induced silencing complex (miRISC) (Bahubeshi et al. 2011). Given the effect that even a single error may have on the miRNA function, it is not unexpected that the interruption of the miRNA manufacturing process has been linked to a diverse array of human malignancies (Foulkes et al. 2014) such as lung, ovarian, and prostate. Dicer1 was discovered to be a significant factor in determining the patient prognosis and the best course of treatment, and its role in carcinogenesis is drawing increasing attention (Karube et al. 2005; Merritt et al. 2008; Chiosea et al. 2006; Conlon et al. 2015). Recent studies have demonstrated an underlying link between cancer stemness and Dicer1. For instance, colorectal cancer cells that exhibited decreased Dicer1 function displayed heighted properties of stemness and elevated levels of metastatic behavior (Iliou et al. 2014).

Let-7 family miRNAs can inhibit cancer growth by limiting cell division (Johnson et al. 2005; Lee and Dutta 2007; Sun et al. 2016). The let-7 group dominates over the stem cell-like traits of BT-ICs in breast cancer through the inhibition of H-RAS and HMGA2 (Yu et al. 2007). Around 2% of endometrial cancers were found to have mutations in the Dicer1 hotspot region, according to recent research. In reaction to Dicer1 mutations, there is a significant increase in the expression of multiple genes, including various members of the let-7 family (Chen et al. 2015). The influence of Dicer1 and cancer stem cells goes beyond mere manipulation of cell mobility and lifespan. In one investigation, they examined the cause of endometrial cancer stemness, focusing on the function of Dicer1 and the let-7 family (Wang et al. 2017b). Clinical samples were profiled for Dicer1 expression, and the relationship between Dicer1 and clinical markers or traits related to stem cells was investigated. In both laboratory settings and living organisms, it was demonstrated that the absence of Dicer1 resulted in elevated characteristics of tumor stemness and aggressiveness. The mechanism behind this tumor-predisposing phenotype was found to be that loss of Dicer1 resulted in aberrant let-7 family expression, and regulated stemness in endometrial cancer cells (Wang et al. 2017b).

The initial step taken by the endoribonuclease Drosha involves converting pri-miRNAs into 70 nt pre-miRNAs through transcription, followed by additional processing. The protein Exportin 5 (XPO5) is responsible for moving pre-microRNAs into the cytoplasm, where it is then taken over by another protein called DICER1 for further processing. Within DICER1, the hairpin structure of the pre-microRNA is cut by a pair of its own RNase III domains, releasing two separate miRNAs from the 3' and 5' ends of the hairpin. It leads to the creation of a miRNA duplex (He and Hannon 2004; Anglesio et al. 2013). The "guide sequence" attaches itself to specific messenger RNAs, whereas the "passenger sequence," often flawed, only partially joins the miRISC complex. People with a genetic mutation in the DICER1 gene are at a higher risk for cancer, particularly familial pleuropulmonary blastoma (PPB) which is more prevalent in individuals with this genetic variation ( (Bahubeshi et al. 2011 Hill et al. 2009)). In addition, individuals with partially differing genetic makeup in their sexual reproductive cells are correlated with the advancement of Sertoli-Leydig cell tumor, multinodular goitre, familial tumor-dysplasia syndrome related to PPB, embryonal rhabdomyosarcoma in the cervix, cystic nephroma, Wilms' tumor, and pineoblastoma (Rio Frio et al. 2011; Foulkes et al. 2011; Doros et al. 2012; Sabbaghian et al. 2012). Recent research has revealed that DICER1 lacks the ability to generate functional 5p miRNAs due to mutations in key areas, specifically the RNase IIIb domain's four metal-binding sites (D1810, E1813, E1705, and D1709) (Heravi-Moussavi et al. 2012; Anglesio et al. 2013; Gurtan et al. 2012). Recent revelations of hotspot tumors linked to DICER1 in both PPB and PPB-FTDS patients who possess hereditary DICER1 mutations offer compelling support for the two-hit theory of cancer development (Heravi-Moussavi et al. 2012; Wu et al. 2013; Tomiak et al. 2014; Kock et al. 2013; Pugh et al. 2014).

The domain of DICER1 RNase IIIb contains four sites where the metal can bind, and these sites have been found to contain frequent mutations in ovarian sex cord-stromal tumors. These same mutations have also been found in other types of pediatric tumors. (Chen et al. 2015). For example, when Chen et al. investigated 290 endometrial cancer samples for DICER1 RNase IIIb domain mutations, they found 6 hotspot changes, including 2 occurrences of an uncommon G1809R mutation adjacent to a metal-binding site (Yi et al. 2003). Utllizing Sanger and Illumina targeted resequencing, they identified and confirmed a biallelic DICER1 mutation in a number of individuals who had hotspot mutations. By using deep sequencing of small RNAs, real-time PCR, and in vitro DICER1 cleavage studies, they showed that the mutations at the metal-binding sites and the alterations at residue 1809 that added a positively charged amino acid side chain had a negative impact on the synthesis of the 5p miRNA. In hotspot-mutated tumors and cell lines, 5p miRNA levels were shown to be generally lower. De-repressed genes caused by loss of 5p miRNAs were shown to be associated with cell cycle regulatory circuits, as determined by pathway analysis of gene expression patterns. Expression of DICER1 hotspot mutations was shown to increase cell proliferation, while expression of wild-type DICER1 suppressed cell proliferation in a mouse cell line deficient in Dicer1. The presence of let-7-targeted genes was more prominent among the up-regulated genes, suggesting that the absence of let-7 may impact subsequent pathways. Their study showed that mutations in DICER1 were linked to several types of cancer and may represent a novel carcinogenic pathway (Chen et al. 2015).

#### Let-7 function

Each stage of mitosis is initiated and progressed by the serine/threonine kinase called Aurora-B. Aurora-B promotes development of chromosomal segregation, cytokinesis, and mitotic spindle formation (Fu et al. 2007). Research has demonstrated that Let-7a is capable of suppressing endometrial cancer through its impact on Aurora-B. Additionally, it is understood that Aurora-B plays a critical role in the efficacy of taxane chemotherapy for non-small cell lung cancer. (Liu et al. 2013; Al-Khafaji et al. 2017). Both let-7c and Aurora-B have been linked to chemosensitivity Paclitaxel primarily works to treat endometrial cancer by causing apoptosis and blocking the breakdown of microtubules in the cells. (Sato et al. 2020). In their study, Sato et al. exposed USPC1 human ESC cells to paclitaxel for 12 weeks, resulting in the creation of two paclitaxel-resistant cell lines (Sato et al. 2020). During 12 weeks of exposure to escalating doses of paclitaxel, two independent cell lines of cells were obtained both showing drug resistance (USPC1-PTXR2 and USPC1-PTSR1). In a microarray study, eight putative microRNAs were found to be potential mediators of paclitaxel sensitivity in USPC1 and USPC1-PTXR1 cells. The paclitaxel-resistant USPC1-PTXR1 cells showed a significant boost in paclitaxel-induced cell death when treated with the let-7c precursor, particularly among cells that were close to the end of their lifespan. On the other hand, the paclitaxel-mediated induction of apoptosis was reversed by suppressing Let-7c. Let-7a is responsible for Aurora-B inhibition, thus inhibiting the development of endometrial cancer cells, by affecting a protein necessary for mitotic progression. Therefore, they investigated whether let-7c also affected Aurora-B expression in ESC cells. USPC-PTXR1 cells expressed more Aurora-B mRNA and protein than USPC1 cells did. After Let-7c was suppressed, In USPC1 cells, the levels of Aurora-B expression were raised, while in USPC1-PTXR1 cells, they were reduced. This indicates that let-7c is responsible for inhibiting the expression of Aurora-B in ESC (endometrial carcinosarcoma) cells, resulting in resistance to paclitaxel. (Sato et al. 2020).

#### Experimental models

Endometrial cancers usually show p16 overexpression as well as TP53 mutations or loss of heterozygosity (Lax 2007; D’Angelo and Prat 2010). Certain cases have also been linked to mutations in other oncogenes including PIK3CA and KRAS (Growdon et al. 2011). Malignant epithelial endometrial canrinoma cells undergo transdifferentiation during the EMT. This process includes the decrease in the expression of E-cadherin (CDH1) and an increase in the expression of N-cadherin and mesenchymal markers like vimentin, fibronectin, and SPARC (an ECM protein). In other EMT models, "cadherin switching" (Wang and Zhou 2011) has been documented along with increased mesenchymal markers; this combination seems to promote invasion and metastasis (Zeisberg and Neilson 2009). SNAI1 also known as ZEB2 (zinc finger E-box binding homeobox 2), as well as ZEB1, E2-2 (as also known as transcription factor 4) and Twist (Castilla et al. 2011), were all up-regulated in ECSs, similar to other tumor types (Wang and Zhou 2011; Peinado et al. 2007; Moreno-Bueno et al. 2008). The molecular and morphological changes in ECSs have been linked to suppressed expression of a few specific miRNAs, for instance miR-203 and miR-200 family (Castilla et al. 2011). Researchers found that ECSs exhibit a significantly increased amount of the AT-hook 2 high mobility group protein (HMGA2), which is a nuclear factor present during embryonic development. This protein specifically binds to areas of DNA that are rich in AT nucleotide base pairs (Reeves et al. 2001; Sgarra et al. 2004) Conformational changes occur, which then modulate transcription. Compared to embryonic tissues, normal adult tissues express HMGA2 at a lower level, whereas HMGA2 is frequently up-regulated in cancers and transformed cells (Sgarra et al. 2004). The connection between HMGA2 and transcription factors that support EMT, such as ZEB1, ZEB2, Snail, and Slug, has also been established (Montserrat et al. 2012). In a variety of tumour types where HMGA2 is overexpressed, members of the let-7 miRNA family can inhibit the expression of HMGA2 (Park et al. 2007). The suppression of this process is connected to the excessive expression of Lin28B, which is responsible for regulating the development of miRNA (Heo et al. 2008). Romero-Pérez et al. utilized cDNA microarrays to analyze the gene expression patterns of 23 samples of endometrioid endometrial carcinoma and compared them to 15 samples of endometrial carcinosarcomas (Romero-Pérez et al. 2013). They found that endometrial sarcomas altered the expression of EMT-related genes, muscle growth, affected the immune response, and level of cancer-testis antigens. Endometrial carcinosarcomas have been shown to express genes that can control the EMT switch during embryonic development, such as HMGA2. Quantitative qPCR and immunohistochemistry were used to confirm the overexpression of HMAG2 in 54 endometrial sarcoma samples. Additionally, they found that let-7b and HMGA2 expression were strongly negatively correlated, suggesting that let-7b has an inhibitory effect on HMGA2 expression. These changes were connected to an increase in Lin28B expression. Only 3% of endometrioid endometrial carcinomas have excessively high levels of the HMAG2 expression, while it is highly expressed in 46% out of 28 nonendometrioid carcinoma samples. This restricted expression pattern in endometrial carcinosarcomas and nonendometrioid carcinomas supports a function for HMGA2 in the establishment of an aggressive phenotype in endometrial carcinoma. It has been proposed that HMAG2 could be a biomarker to identify patients whose cancers are with or without endometrioid characteristics (Romero-Pérez et al. 2013).

**Table 3:** Table 3, Shows various research that specifically  addresses the significance of let-7 in the development  of endometrial cancer.

Expression status	Target	Model	Type of cell line	Ref
Downregulated	Hmga2, c-Myc, and Igf2bp3	In vivo (tissue) and In vitro	A2780, Tara R127, ARK2, and HEK29314	Yan et al. 2015)
Downregulated	-	In vivo and In vitro	Ishikawa	Wang et al. 2020)
Downregulated	H-RAS and HMGA2	In vivo (tissue) and In vitro	RL-95, HEC-1B, An3ca, and Ishikawa	Wang et al. 2017b)
Downregulated	Aurora-B	In vitro	USPC1, USPC1-PTXR, and USPC1-PTXR2	Sato et al. 2020)
Downregulated	HMGA2	In vitro	HEC1A, SK-UT-1 and SK-UT-1B	Romero-Pérez et al. 2013)
Downregulated	CDC25A, CCNA2, CCNE1, CDK1, SKP2, and LIN28A/B	In vitro	DKO172	Chen et al. 2015)
Downregulated	Aurora-B	In vitro	HeLa and Ishikawa	Liu et al. 2013)
Upregulated	BAX	In vitro	Ishikawa, ECC-1, and HEC-1B	Zhang et al. 2012)
Downregulated	Hmga2, c-Myc and Igf2bp3	In vivo (tissue) and In vitro	A2780, Tara R127, ARK2, and HEK293	Yan et al. 2015)

## Exosomal let-7 and gynecological *cancer*

**Fig. 6:**
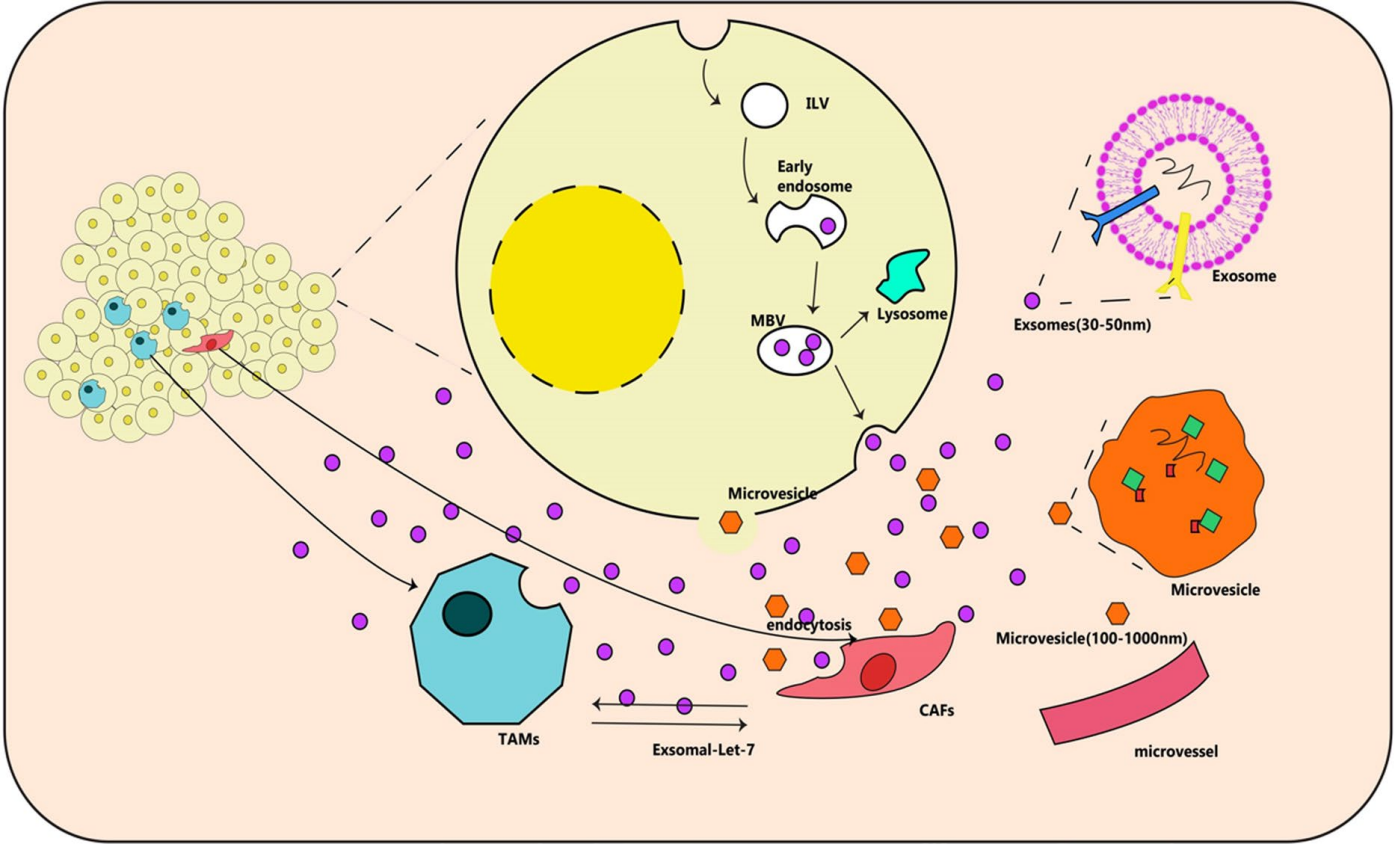
The secretion of extracellular vesicles (EVs) involves several distinct mechanisms. One such mechanism is the direct formation of microvesicles (MVs) through the budding of the plasma membrane. A different process includes changing extracellular vesicles (EVs) into intraluminal vesicles (ILVs) while they are inside multivesicular endosomes (MVBs), and subsequently letting them out by merging with the cell's outer layer, eventually creating exosomes. The essential function of these exosomes is to aid in the transmission of messages between tumor cells and other types of cells, including tumor-associated macrophages (TAMs) and cancer-associated fibroblasts (CAFs). The exosomes contain important molecules, including let-7 and others, which are transferred to various cells through both microvessel transport and direct internalization by TAMs, CAFs, and other cell types

A recent study has brought attention to the potential of tissue-specific, small-sized vesicles known as exosomes in detecting diseases early on and continuously monitoring treatment (Taylor and Gerçel-Taylor 2005; Simpson et al. 2008; Zhang et al. 2023; Rahbaran et al. 2022). Exosomes are endocytic membrane vesicles with nanoscale dimensions (Denli et al. 2004; Landthaler et al. 2004; Alarcón et al. 2015; Han et al. 2004; Yi et al. 2003; Bohnsack et al. 2004; Lund et al. 2004; Lee et al. 2016; Reichholf et al. 2019; Nam et al. 2011; Heo et al. 2012; Patterson et al. 2017; Mo et al. 2019; Brameier et al. 2011; Berezikov et al. 2007; Westholm and Lai 2011; Salim et al. 2022; Babiarz et al. 2008; Chong et al. 2010; Cifuentes et al. 2010; Yang et al. 2012a; Yi et al. 2015; Herrera-Carrillo and Berkhout 2017; Dupuis-Sandoval et al. 2015; Kufel and Grzechnik 2019; Meier 2017; Li et al. 2011; Ender et al. 2008; Scott et al. 2009; Macias et al. 2012; Taft et al. 2009; Langenberger et al. 2013; Macias et al. 2015; Falaleeva et al. 2016; Abdelfattah et al. 2014; Hasler et al. 2016; Li et al. 201; Reinsborough et al. 2019; Kumar et al. 2014; Kuscu et al. 2018; Zhang et al. 2018a; Gagnon et al. 2014; Robb et al. 2005; Rüdel et al. 2008; Nishi et al. 2013; Yoon et al. 2015; He and Schneider 2006; Yoon et al. 2013; Karege et al. 2010; Correa et al. 2012; Peart et al. 2012; Manning and Toker 2017; Linnerth-Petrik et al. 2016; Balasuriya et al. 2018; Balasuriya et al. 2020; Alessi et al. 1997; Sarbassov et al. 2005; Gao et al. 2005; Kuo et al. 2008; Wei et al. 2017; Que et al. 2015; Fan et al. 2022; Wang et al. 2012a; Frederick et al. 2022; Gupta et al. 2019; Byrum et al. 2019; Coleman et al. 2017; Moore et al. 2019; González-Martín et al. 2019; Mirza et al. 2016; Poveda et al. 2021; Falzone et al. 2021; Jiang et al. 2019; An et al. 2021; Chirshev et al. 2020; Zhou et al. 2013; Parisi et al. 2017; He et al. 2018; Albino et al. 2016; Navarra et al. 2016; Wang et al. 2018; Brueckner et al. 2007; Shi et al. 2017; Wielgos et al. 2017; Huang et al. 2017; Shen et al. 2014; Chirshev et al. 2021; Hoppenot et al. 2018; Konecny et al. 2014; Coleman et al. 2013; Tothill et al. 2008; Tan et al. 2013; Bedard et al. 2013; Vaughan et al. 2011; Beck and Blanpain 2013; Agarwal and Kaye 2005; Verhaak et al. 2013; Grosse-Wilde et al. 2015; Strauss et al. 2011; Hojo et al. 2018; Lu et al. 2012a; Kurrey et al. 2009; Yang et al. 2008a; Yang et al. 2012b; Wang et al. 2013; Sorrentino et al. 2008; Hayes and Ruvkun 2006; Unternaehrer et al. 2014; Yang et al. 2008b; Iorio and Croce 2013; Zhang et al. 2015) (Fig. 6). Exosomes undergo a biosynthetic process that begins with the inward creation of small pockets within multivesicular bodies (MVBs), which contain RNAs and proteins (Shao et al. 2021; Liang et al. 2022; Yao et al. 2023; Lu et al. 2022). A variety of cells, including cancer cells, release exosomes and, they can be found in different bodily fluids, such as blood, urine, saliva, and breast milk, and can also be identified in the outer region of the body (Lässer et al. 2011; Alvarez et al. 2012). It has been indicated by multiple research that exosomes released by tumors might influence the disease development. Exosomes released by some tumor cells may spread oncogenic properties to other tumor cells (Karami Fath et al. 2022). Exosomes are a useful source of cancer biomarkers because they are: (i) actively secreted from living cancer cells; (ii) contain information about the tumor conditions; (iii) can be easily retrieved from biological fluids; (iv) provide information that is easily extracted from high-abundance proteins; (v) are extremely stable. Importantly, exosomes are secreted by actively proliferating tumor cells and are different from apoptotic cell bodies (Théry et al. 2001). Exosomes carry cell-type-specific RNA and protein molecules they also, may reveal a great deal about the tumor hallmarks. There are many different cell surface receptors and proteins that exosomes have been identified to contain. Some examples are heat shock proteins, adhesion molecules, cytoskeleton proteins, and proteins related to fusion and membrane movement. Moreover, exosomes contain miRNA molecules, which may affect the interactions between donor and recipient cells (Ambros 2004). It is not yet completely clear how exosomes contribute to cancer development and metastasis (Kobayashi et al. 2014). Current research lends credence to the idea that exosomes released by host cells might alter the immediate extracellular environment to favor tumor cell proliferation and neovascularization. Exosomes may also alter the parent cell phenotype or the phenotype of the recipient cells as a type of intercellular communication. Cell signaling molecules may become trapped in exosomes, resulting in decreased availability within the cell and potentially changing the cell's appearance and ability to spread to other parts of the body. Exosomes may potentially alter cellular behavior and function once they fuse with their intended target cell.

One study evaluated the miRNAs contained in exosomes released by two ovarian cancer cell lines with distinct invasion capacities. The main goal of this research was to investigate whether there is a connection between the release of exosomes and the sequestration of miRNA in ovarian cancer cells and their ability to invade surrounding tissue. Analysis was done on the exosome release characteristics of ovarian cancer cell lines, low invasion SKOV-3 and high invesion OVCAR-3. The viability SKOV-3 of and OVCAR-3 cells was assessed after 24 h of cultivation in a DMEM supplemented with 20% exosome-free FBS containing 8% O2. The exosomes were extracted from the conditioned media of cells through the process of differential and buoyant density centrifugation. The identity of exosomes was verified through Western blotting, which detected the presence of specific markers such as CD63 and CD9. The expression of miR-200a-c and Let-7a-f, two miRNAs identified in exosomes, were quantified using real-time PCR. The cup-shaped round morphology of the vesicles and the CD63 and CD9 expression confirmed their identity as exosomes. By comparing the exosome secretion of SKOV-3 and OVCAR-3 cells, they found that the former cells produced 2.7 times more exosomes (1.22 0.11 g/106 cells) than the latter. Exosomes and ovarian cancer cell lines both contained let-7 family mRNAs. Let-7 family transcript levels in OVCAR-3 cells were higher in comparison to those in SKOV-3 cells. On the other hand, SKOV-3 exosomes contained more let-7 family transcripts in comparison to OVCAR-3 exosomes. It was discovered that only OVCAR-3 cells and their exosomes contained miR-200 family transcripts. Therefore, exosomes secreted by various ovarian cancer cell lines significantly have different, capability for invasion (Kobayashi et al. 2014).

Exosome research is one of the most crucial applications of liquid biopsies in precision medicine (Denzer et al. 2000). Exosomes are capable of transferring various molecules such as lipids, proteins, DNA, and various types of RNA, including messenger RNA, long non-coding RNA, and short RNAs. They play a significant role in the progression and dissemination of cancer (Wang et al. 2016). Mature detection techniques have been developed for exosomal miRNAs, because they are interesting diagnostic markers for cancer and other diseases since they are long-lived and relatively non-degradable. In contrast, cell-free DNA possesses heterogeneous mutation sites, lncRNAs have complex biological activities, and mRNA is very unstable (Taylor and Gercel-Taylor 2008). The use of exosomal miRNAs has been proven to be valuable in identifying cancer and tracking its progression, making them effective biomarkers for diagnosis and early detection. The severity of prostate tumors can vary greatly, with some being relatively harmless while others are life-threatening. However, by utilizing a five-miRNA gene signature, it may be possible to differentiate between these different types of tumors (Alhasan et al. 2016). MiR-122, miR-192, miR-17-5p, and miR-25-3p are known to be abundant in various cancers, in addition to being highly concentrated in tumor-derived exosomes (Wang et al. 2009). Detecting the levels of miRNA in the blood, specifically in serum or plasma, serves as a means of identifying non-invasively the presence of squamous cell carcinoma (SCC) in the cervix, both prior to and following surgery (Denzer et al. 2000), as well as for non-small cell lung cancer (Jin et al. 2017). A research project examined the characteristics of exosomal miRNAs as potential diagnostic indicators, utilizing a sample size of 121 plasma samples from individuals without any significant health concerns, those with cervical cancer, and patients diagnosed with CIN (Zheng et al. 2019). Researchers found a group of eight exosomal miRNAs that had varying levels of expression in individuals with CIN I and in healthy individuals. These miRNAs were also able to effectively differentiate between patients with advanced CIN II and those with early lesions, demonstrating their potential as biomarkers. They discovered that miR-30d-5p and Let-7d-3p showed a consistent expression pattern in plasma samples, as confirmed in 203 different plasma samples, and were significantly different between cervical tumors and surrounding normal tissues (P 0.005). These two miRNAs taken together provided an AUC of 0.828 for distinguishing between CIN II + and CIN I- patients. The AUC increased from 0.766 to 0.887 when combined with cytological testing. In conclusion, the diagnostic biomarkers miR-30d-5p and let-7d-3p plasma exosomes could be used to detect cervical cancer and its precursor lesions at an early stage without the need for invasive procedures (Zheng et al. 2019).

The prevalence of the let-7 miRNA cluster is apparent in the extracellular elements, particularly in the exosomes released by AZ-P7a cells. This suggests that these miRNAs may actively contribute to the progression and dissemination of cancer cells. The research conducted by Ohshima et al. indicates that AZ-P7a cells utilize exosomes to release let-7 miRNAs, which are typically known for their ability to inhibit tumor growth by targeting oncogenes such as RAS and HMGA2. This suggests that these cells employ this method to maintain their oncogenic actions and contribute to the development of cancer (Ohshima et al. 2010). It has been proposed that EVs containing let-7 can impact various pathways and targets within recipient cells, potentially influencing viral replication, the human body's ability to fight against viruses, and the activity of viruses associated with cancer. Most organisms experience an increase in let-7 levels as they reach the later stages of development, and altering its expression can potentially lead to the emergence of various pathological conditions such as neurodegenerative diseases, cancer, and diabetes (Shamsuzzama et al. 2016). The Let-7 molecule plays a role in regulating cellular signaling pathways. Overexpression of miR-98 leads to inhibition of these pathways, specifically suppressing the Akt and ERK pathways, which have been linked to cancer development. Researchers discovered that in Ewing's sarcoma, the presence of let-7 ultimately led to a significant decrease in the levels of signal transducer and activator of transcription 3 (STAT3), which ultimately resulted in a reduction of the cancer's aggressive traits. The STAT3 pathway plays a critical role in controlling the cell cycle and promoting cell viability through targeted genes. Thus, excessive activation of this pathway can propel the advancement of cancer. The activation of the STAT3 signaling pathway has been associated with unfavorable treatment results and heightened resistance towards chemotherapy and radiation (Zhang et al. 2016). Recent research has indicated that Let-7 functions to stimulate the WNT signaling pathway in breast cancer by directing its attention to estrogen receptors. Furthermore, in hepatocellular carcinoma (HCC), Let-7 has been found to target transcription factor 4 (TCF-4), a key member of the WNT pathway. This leads to an upregulation of cancer stemness and aggressive behavior (Chirshev et al. 2019). The WNT pathway plays a crucial role in a number of cellular processes, including differentiation, proliferation, and migration. Its activation has been associated with a rise in tumor growth and the formation of cancer stem cells. Research suggests that let-7 can counteract the aggressive behavior of cancer by inhibiting the activity of pathways that promote cancer development (Letafati et al. 2022).

**Table 4:** Table 4 contains a compilation of various research articles that have investigated the function of exosomal let-7 in gynecological cancer.

Diseases	Expression	Target	Model	Type cell line	Ref
Ovarian cancer	Upregulated	CD63 and CD9	In vitro	SKOV-3 and OVCAR-3	Kobayashi et al. 2014)
Cervical cancer	Upregulated	adherens junction, hippo signaling pathway, cell cycle, p53 signaling pathway, AMPK signaling pathway	In vivo	-	Zheng et al. 2019)
Cervical cancer	Upregulated	p53	In vitro	HeLa, SiHa	Honegger et al. 2015)

## Let-7 as a tool for therapy

One commonly used approach for assessing the impact of miRNA gain or loss of function involves the use of chemically modified oligonucleotides, which actively bind to or reintroduce miRNAs. The first instance of an in vivo miRNA inhibitor was demonstrated by injecting a 2′-O-methylated oligonucleotide, which was a matching sequence to let-7, into *C. elegans*. This resulted in a phenotype that indicated a loss of function in the larvae (Hutvágner et al. 2004). As an improvement, to enhance the efficiency of communicating with the cell membrane, changes were made to 2′-O-Me oligonucleotides by incorporating a 3′ cholesterol element, known as antagomirs. The initial experimentation involved administering miR-16 through intravenous injection to healthy mice (Krutzfeldt et al. 2005). Using an anti-let-7 2′OMe oligonucleotide to chemically inhibit the action of let-7 miRNA solidified the evidence that this particular miRNA is capable of suppressing cell proliferation pathways in human cells (Johnson et al. 2007b). Based on the available evidence, multiple treatment methods have been developed to focus on either targeting or reintroducing let-7 for the purpose of therapy. The group offered groundbreaking evidence that the let-7 molecule plays a crucial role in the development of cancer. Their research on C. elegans revealed that the let-7 miRNA family regulates the function of RAS and pinpointed specific let-7 locations in the 3′UTRs of RAS in both human cells and lung tissue. This demonstrates, once again, that the let-7 miRNA family effectively hinders RAS activity in both *C. elegans* tissues and human cells, serving as the first recorded instance of a miRNA acting as a cancer suppressor (Johnson et al. 2005).

Subsequently, it was found that let-7 is linked to lung cancer through the discovery that introducing let-7g into lung cancer cells with overactive Kras led to the suppression of cellular proliferation and induction of cell death. In a broader sense, all members of the let-7 family are disrupted in cancer, with most being reduced in expression. Furthermore, the lack of let-7 family members points to a negative outlook in several forms of cancer (Johnson et al. 2005). Various novel techniques have been employed in cancer treatment to harness the tumor-suppressing power of let-7, such as systemic mimic delivery via neutral lipid emulsions to reintroduce let-7 into lung tumors. This method demonstrated the successful delivery of let-7b throughout the body, leading to its concentration in the tumor and resulting in inhibitory effects on lung cancer growth in a mouse model with the KRASG12D genetic mutation (Trang et al. 2011).

Reintroducing the let-7b mimic has been scientifically demonstrated to successfully suppress the cancer-causing gene c-MYC and effectively counteract the resistance to multiple drugs in gastric cancer. In a similar manner, the use of let-7 has been noted to enhance the response to chemotherapy in a cell model with a mutated KRAS gene (Dai et al. 2015; Yang et al. 2015). In a notable development, there have been multiple strategies aimed at effectively targeting the main upstream factors of let-7 for therapeutic purposes. A significant strategy lies in directing attention towards Lin28b, a well-established controller of let-7 production and buildup. This method has been employed in treating pancreatic cancer, where reducing the levels of the oncogenic protein Lin28b by utilizing SIRT6 resulted in elevated levels of the let-7 family member. This approach effectively hindered the growth of PDAC cells in experimental models (Gilles and Slack 2018).

## Clinical performance of let-7

The Let-7 family is made up of tiny non-coding RNA molecules that oversee the expression of numerous genes responsible for crucial cellular functions (379, 380). Nevertheless, this will necessitate meticulous examination and additional retrospective studies, followed by rigorous clinical trials. The primary goal of the investigation conducted by Lu and colleagues was to analyze the correlation between the degree of let-7a expression and the likelihood of survival among individuals with epithelial ovarian cancer (EOC) who underwent different chemotherapy regimens (Lu et al. 2011b). A total of 178 epithelial ovarian cancer (EOC) patients who had undergone platinum-based chemotherapy with or without paclitaxel following surgical intervention were included in the study. The levels of let-7a were evaluated via qRT-PCR and its impact on the progression of diseases was explored using survival analysis. Although present in EOC specimens, the presence of let-7a was not observed to have any correlation with the severity of the disease, grade of the tumor, type of tissue, or the effectiveness of debulking. It was noted that individuals who showed a positive reaction to platinum chemotherapy, in combination with paclitaxel, exhibited considerably reduced quantities of let-7a compared to those who did not experience a favorable response. Upon further examination, it was revealed that individuals with elevated levels of let-7a experienced superior rates of survival when given platinum treatment alone, in contrast to those with depleted let-7a levels. The likelihoods of mortality and disease advancement were significantly lower, at 0.52 and 0.48 respectively, in those with high let-7a levels. In contrast, administering platinum and paclitaxel to patients led to unfavorable outcomes in those with elevated levels of let-7a, as indicated by their significantly lower progression-free and overall survival rates with hazard ratios of 3.87 and 3.48, respectively. Further studies showed that patients with lower levels of let-7a had improved survival results when treated with both paclitaxel and platinum, compared to those treated solely with platinum. On the other hand, in individuals who have elevated levels of let-7a, the particular type of treatment received did not have a notable impact on their survival rates. This reinforces the idea that the favorable outcome of incorporating paclitaxel in the treatment of EOC is closely tied to the presence of let-7a, underscoring the value of miRNAs like let-7a as valuable indicators for selecting suitable chemotherapy options in managing EOC (Lu et al. 2011b).

Wu et al. (Wu et al. 2015b) demonstrated that a decrease in let-7a expression in primary breast tumors was linked to a resistance to epirubicin. Additionally, In a controlled laboratory environment, the amplification of let-7a expression caused breast cancer cell lines that were initially resistant to epirubicin to become more vulnerable to the drug through the stimulation of cell death. These findings indicate that lower levels of let-7a microRNA may contribute to resistance against chemotherapy in breast cancer through the inhibition of apoptosis, and propose the consideration of let-7a as a potential means of combating resistance to epirubicin-based chemotherapy (Wu et al. 2015b).

## Limitations and future perspective of Let-7–based therapy

### Limited knowledge of Let-7 biology

The lack of complete understanding about the regulation and function of let-7 during biogenesis and in the development of tumors poses challenges in using it as a direct therapy, despite its beneficial effects on restoring normal expression. It is crucial to determine whether the decrease of let-7 in tumors is a primary event or a result of other factors. Based on the CSC theory, the process of epigenetic suppression of let-7 in cancer stem cells leads to the increased activity of oncofetal genes, such as HMGA2 and LIN28, causing a lack of specialization and the development of tumors. The initial occurrence of let-7 being reduced is reinforced by the discovery that let-7 is excessively methylated in ovarian cancer. Additionally, the effectiveness of let-7 as a therapeutic needs to be evaluated, as truncated 3′ UTR oncogenes may be prevalent in neoplastic cells and could affect the function of let-7. Grimm et al. (Grimm et al. 2006) reported the administration of recombinant pre-mirnas using adeno-associated virus resulted in fatalities in mice due to extreme liver toxicity. More research projects are needed to fully understand the immunogenic and cytotoxic effects of let-7 and develop effective treatment measures to minimize these adverse reactions. It has been suggested that let-7 may have a remarkable effect on regulatory network of miRNAs, similar to the network involving transcription factors, but the exact mechanisms are still unknown. As a result, the use of anti-mirna oligos to target let-7 inhibitory mirnas is not currently feasible.

### Delivery methods and systems

One major drawback of potential let-7 treatment is the absence of an appropriate, reliable, and effective method of delivery. It is crucial to establish a standardized approach for the utilization of biological carriers such as aav and lentivirus in order to avoid unintended introduction into non-targeted locations, despite their potential for targeted delivery. Additionally, the delivery of brain-specific mirna remains unsuccessful and specialized approaches for neuron-specific delivery must be devised to address brain and neuronal tumors. As noted earlier, the utilization of AAV and lentivirus as vehicles for administering let-7 in a mouse model of lung cancer proved to be unsuccessful as the tumor developed resistance to let-7 over time. Consequently, an approach for implementing let-7 treatment in pre-existing tumors must be devised (Barh et al. 2010).

### Future potential strategies to overcome the limitations

The ideal or typical level of let-7 may be reinstated in cancer cells by either introducing external let-7 through a vector that amplifies let-7 or suppressing let-7 inhibitors. Recent progress in the field of miRNA technology has primarily centered on the utilization of complementary or altered single-stranded RNA molecules (or a combination thereof) to inhibit targeted miRNAs that are implicated in diseases or cancer. These altered versions, including antisense oligonucleotides (ASOs), anti-Mirna ASOs (known as "antagomirs"), locked nucleic acids, and antisense technology-based small interfering RNAs, have proven to be highly effective in regulating miRNA activity.However, there is scarce data on the miRNAs responsible for controlling let-7 expression, which limits the potential of utilizing this strategy. As a result, alternate techniques that can efficiently boost let-7 expression must be employed. Thus, the utilization of a vector to amplify let-7 expression or the short-term introduction of double-stranded let-7 seems to be the most viable solution. This approach has the potential to generate mature let-7, analogous to the natural form, through the Dicer process, thus effectively restoring a diminished let-7 level. The effectiveness of this method has already been demonstrated. It is possible to achieve high levels of let-7 expression by using vectors containing synthetic short hairpin RNAs that mimic pre-let-7 and are activated by inducible Pol III promoters such as H1 and U6. However, a more effective approach may be to insert the complete natural pri-let-7 hairpin, along with its flanking sequences, into the expression vector rather than creating artificial hairpins. This is based on the assumption that the natural pre-let-7 structure will be more suitable as a substrate for generating mature let-7 during the Dicer processing. The successful use of a pri-mir-Pol II transgene system has allowed for the efficient overexpression of miR-155, miR-30, and miR-122. It has also been observed that this system has the ability to effectively express multiple microRNAs from a single transcript, making it a viable choice for achieving let-7 expression. Additionally, a delivery method involving the combination of high-density lipoprotein and siRNAs has demonstrated improved effectiveness in targeting specific organs such as the liver, gut, kidney, and steroid-producing organsA comparable strategy may also be effective in administering let-7. Yet, producing and purifying high-quality let-7 for therapeutic use is a significant challenge. Utilizing nanoparticles as a delivery mechanism may prove advantageous. Other delivery techniques, including administering lentivirus-mediated oligonucleotides containing pre-let-7, introducing mature let-7 hairpin sequences via adenovirus, delivering pre-let-7 through cationic liposomes, and using synthetic let-7 with electroporation, have demonstrated promising results in both laboratory and animal studies. While these approaches are still in the developmental stage, they have the potential to become viable therapeutic options in the near future. (Barh et al. 2010).

## Conclusion

A variation in miRNA levels has been associated with numerous illnesses such as cancer, inflammatory diseases, cardiovascular issues, and gynaecological disorders. The let-7 microRNA family, found in a wide range of cells, plays a crucial role in the differentiation process of multiple cell types during embryonic development. In addition, there is an clear relationship between the downregulation of let-7 and poorly differentiated aggressive tumors including gynecological cancers. There are various levels of control over let-7 expression, and some types of gynaecological cancer show dysregulation of certain let-7 group members. However, it is still unclear if there are actually 13 distinct members of the let-7 family, because they have different targets and there may be some overlap, or whether there are many genes that allow for more complex regulation in different cell/tissue types.. There is evidence that tumor resistance to chemotherapy drugs and radiation treatment is related to lower let-7 expression, Although the exact process is not fully understood, it is unknown why let-7 is not considered a factor in developing resistance to medication. Despite some uncertainties, existing research indicates that boosting let-7 levels could be a promising method for treating gynecological cancer patients with reduced let-7 expression.

The aforementioned studies illustrate the biological and mechanistic role of let-7 in the development and progression of GC. However, there are various limitations that need to be addressed before utilizing let-7 in therapy. One major concern is the relationship between the conservation of miRNA sequences and their molecular function in different species. Additionally, the majority of lncRNAs regulate a network of genes rather than a single pathway, particularly for miRNAs involved in epigenetic remodeling. Consequently, manipulating miRNA expression may lead to unforeseen complications. Furthermore, the role of lncRNAs can differ based on the existence of varying forms or modifications of RNA., such as editing or methylation. Additionally, the diverse functions of lncRNAs are influenced by their subcellular localization. It has been observed that the same lncRNA present in different cellular compartments—such as the nucleus, cytosol, or mitochondria—can have distinct roles. Considering the numerous associations between let-7 and the spread of cancer cells as well as the maintenance of stem cells in breast cancer, it is probable that let-7 will be a viable target for treatment in gastric cancer patients. Furthermore, deviations in the levels of let-7 family genes have been connected to various types of cancer, although a more comprehensive comprehension of the exact mechanisms is necessary in order to fully utilize the therapeutic possibilities of microRNAs.

## Data Availability

Not applicable.
